# Revealing the composition-dependent structural evolution fundamentals of bimetallic nanoparticles through an inter-particle alloying reaction†

**DOI:** 10.1039/d1sc06296d

**Published:** 2022-03-25

**Authors:** Yingzheng Lin, Yitao Cao, Qiaofeng Yao, Jianping Xie

**Affiliations:** Joint School of National University of Singapore and Tianjin University, International Campus of Tianjin University Binhai New City Fuzhou 350207 P. R. China; Department of Chemical and Biomolecular Engineering, National University of Singapore 4 Engineering Drive 4 Singapore 117585 Singapore chexiej@nus.edu.sg qfyao@tjufz.org.cn

## Abstract

Alloy nanoparticles represent one of the most important metal materials, finding increasing applications in diverse fields of catalysis, biomedicine, and nano-optics. However, the structural evolution of bimetallic nanoparticles in their full composition spectrum has been rarely explored at the molecular and atomic levels, imparting inherent difficulties to establish a reliable structure–property relationship in practical applications. Here, through an inter-particle reaction between [Au_44_(SR)_26_]^2−^ and [Ag_44_(SR)_30_]^4−^ nanoparticles or nanoclusters (NCs), which possess the same number of metal atoms, but different atomic packing structures, we reveal the composition-dependent structural evolution of alloy NCs in the alloying process at the molecular and atomic levels. In particular, an inter-cluster reaction can produce three sets of Au_*x*_Ag_44−*x*_ NCs in a wide composition range, and the structure of Au_*x*_Ag_44−*x*_ NCs evolves from Ag-rich [Au_*x*_Ag_44−*x*_(SR)_30_]^4−^ (*x* = 1–12), to evenly mixed [Au_*x*_Ag_44−*x*_(SR)_27_]^3−^ (*x* = 19–24), and finally to Au-rich [Au_*x*_Ag_44−*x*_(SR)_26_]^2−^ (*x* = 40–43) NCs, with the increase of the Au/Ag atomic ratio in the NC composition. In addition, leveraging on real-time electrospray ionization mass spectrometry (ESI-MS), we reveal the different inter-cluster reaction mechanisms for the alloying process in the sub-3-nm regime, including partial decomposition–reconstruction and metal exchange reactions. The molecular-level inter-cluster reaction demonstrated in this study provides a fine chemistry to customize the composition and structure of bimetallic NCs in their full alloy composition spectrum, which will greatly increase the acceptance of bimetallic NCs in both basic and applied research.

## Abstract 2

An inter-particle reaction between atomically precise [Au_44_(SR)_26_]^2−^ (SR = thiolate) and [Ag_44_(SR)_30_]^4−^ nanoparticles reveals the composition-dependent structural evolution of alloy Au_*x*_Ag_44−*x*_ nanoparticles at the atomic level.

## Graphical abstract

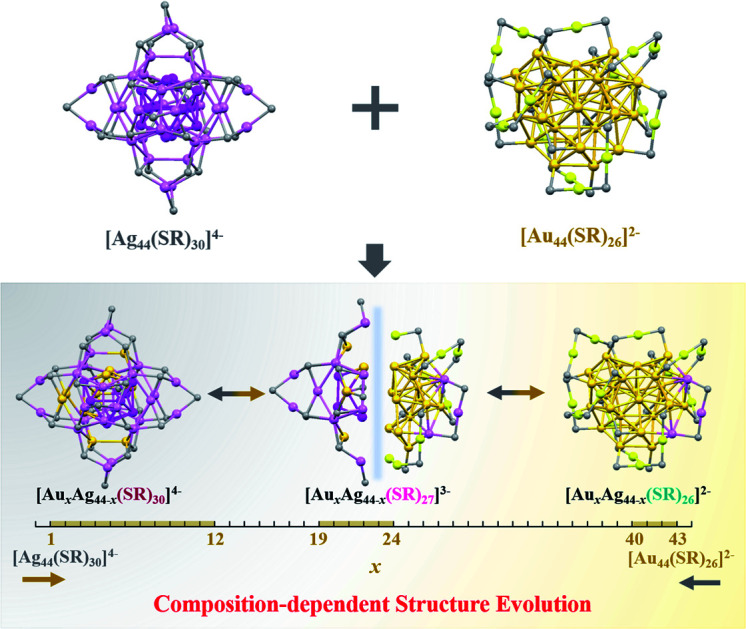

## Introduction

Alloying chemistry has been extensively developed as a versatile and effective means to integrate and synergize the physicochemical properties of different metals, and it has been used in various fields to improve material stability and performance. For example, Cu/Ag binary alloys show combined advantages of high strength and good conductivity, which have been widely used in the electric field.^1,2^ In addition to the remarkable success in engineering bulk metal materials, the prosperity of alloying chemistry has recently propagated into the nanoscale, where the ratio and spatial arrangement of hetero-metal atoms are critical to determining the electronic, optical, and catalytic properties of metal nanomaterials.^3–9^ In other words, determining the packing mode of different metal atoms in alloy materials plays an important role in customizing material properties. The packing mode of alloy materials is highly related to but different from the parent metal materials. Different metal elements show unique packing behaviors in forming bulk or nano-metal materials due to their distinct geometrical and electrical structures. Therefore, in the synthesis of alloy materials with a full composition spectrum, the packing mode evolution of hetero-metal atoms still remains mysterious. However, due to the inherent difficulties in producing plasmonic metal nanoparticles (>3 nm) with extremely high monodispersity, the underlying chemistry governing the alloying process of metal nanomaterials, especially for the structural evolution with composition change, has been rarely revealed.

In the past two decades, atomically precise metal nanoclusters (NCs) have been extensively studied in both basic chemical science and practical applications.^10–13^ Metal NCs are ultrasmall particles with a typical core size of <2 nm. Advances in cluster chemistry in the past two decades have allowed the synthesis of dozens of mono-, bi- and multi-metallic NCs with molecular purity, which make them descriptive with a molecular formula.^14–17^ For example, Au_*x*_Ag_*y*_(SR)_*z*_ denotes an alloy NC consisting of *x* Au atoms, *y* Ag atoms, and *z* thiolate (SR) ligands. The atomically precise structure of metal NCs together with their size-dependent molecular properties (*e.g.*, optical absorption and luminescence) provides an ideal platform for revealing alloying chemistry at the molecular and atomic levels.^18–23^

Metal NCs with an atomically precise structure are ideal model metal nanoparticles for studying the structural evolution of alloy nanoparticles in the composition changing process. For the special case of Au_*x*_Ag_25−*x*_(SR)_18_ NCs, their continuous composition control has been achieved in the full spectrum of *x* = 0–25. For example, an inter-cluster reaction method between Au_25_(SR)_18_ and Ag_25_(SR)_18_ has been recently used to produce Au_*x*_Ag_25−*x*_(SR)_18_ NCs in a full spectrum of *x* = 1–24.^24,25^ In addition to coinage metals, other transition metals (*e.g.*, Ir) can also be doped into Au_25_(SR)_18_ NCs (*e.g.*, Au_22_Ir_3_(SR)_18_) *via* an inter-cluster reaction.^26^ In addition, the reaction of the parental Au_25_(SR)_18_ with the Ag(i) precursors can form Au-rich Au_*x*_Ag_25−*x*_(SR)_18_ NCs,^27–31^ while the reaction of Ag_25_(SR)_18_ with Au(i) precursors can produce Ag-rich Au_*x*_Ag_25−*x*_(SR)_18_ NCs with *x* = 1–7.^32–34^ However, except the ubiquitous alloy NC family of Au_*x*_Ag_25−*x*_(SR)_18_ NCs, in which Au_25_(SR)_18_ and Ag_25_(SR)_18_ adopt the same framework, most of the Au and Ag based NCs with the same metal number show distinct structures. This is probably due to the different coordination habits of Au and Ag atoms, where Ag atoms possess a relatively flexible coordination sphere and thus can form Ag-SR protecting motifs with diverse structures (*e.g.*, mount-like Ag_2_(SR)_5_ motif in [Ag_44_(SR)_30_]^4−^ and staple-like SR-[Ag-SR]_2_ motif in [Ag_25_(SR)_18_]^−^).^35–37^ In contrast, Au atoms prefer the staple-like SR-[Au-SR]_*x*_ motifs ubiquitously in [Au_*n*_(SR)_*m*_]^*q*^ NCs.^38^ Such distinct differences in the coordination habits are well reflected by the crystal structures of [Ag_44_(SR)_30_]^4−^ and [Au_44_(SR)_26_]^2−^, which have the same number of metal atoms but different packing modes. Specifically, [Ag_44_(SR)_30_]^4−^ has a hollow icosahedral Ag_12_-based Ag_32_ core capped by six mount-like Ag_2_(SR)_5_ motifs,^35,36^ while [Au_44_(SR)_26_]^2−^ has a bi-icosahedral-based Au_29_ core capped by two terminal SR, three SR-Au-SR, and six SR-[Au-SR]_2_ motifs.^39^ It is also worth noting that the reported alloy Au_*x*_Ag_44−*x*_ NCs so far exclusively adopted the M–S framework of [Ag_44_(SR)_30_]^4−^, where up to 12 Ag atoms are substitutable by Au atoms.^35,40–42^ Therefore, [Ag_44_(SR)_30_]^4−^, [Au_44_(SR)_26_]^2−^ and their alloy NCs provide an ideal platform for decoding the composition dependent alloying chemistry at the molecular and atomic levels, although an efficient synthetic method should be developed for fine tuning the composition of alloy Au_*x*_Ag_44−*x*_ NCs in a wide composition range.

Here, we report the synthesis of alloy Au_*x*_Ag_44−*x*_ NCs with a wide composition range by an inter-cluster reaction between [Ag_44_(SR)_30_]^4−^ and [Au_44_(SR)_26_]^2−^, which possess an identical number of metal atoms, but a different number of SR ligands and different atomic packing structures. To exclude any possible ligand effects on the structural evolution of alloy NCs, we chose the same *para*-mercaptobenzoic acid (*p*-MBA) as the model thiolate ligand in both parental NCs. Indeed, by simply adjusting the molar ratio of Au_44_ and Ag_44_ NCs (hereinafter referred to as *R*_Au_44_/Ag_44__) in the inter-cluster reaction, the composition of Au_*x*_Ag_44−*x*_ NCs can be fine-tuned within a wide range, *i.e.*, *x* = 1–12, 19–24 and 40–43. More intriguingly, with the increase of the Au content (*i.e.*, *x* value) in Au_*x*_Ag_44−*x*_ NCs, the cluster structure evolves from [Au_*x*_Ag_44−*x*_(SR)_30_]^4−^ (*x* = 1–12), through [Au_*x*_Ag_44−*x*_(SR)_27_]^3−^ (*x* = 19–24), and finally to [Au_*x*_Ag_44−*x*_(SR)_26_]^2−^ (*x* = 40–43), where the intermediate species [Au_*x*_Ag_44−*x*_(SR)_27_]^3−^ NCs (*x* = 19–24) were captured for the first time in this study. Moreover, based on extensive real-time electrospray ionization (ESI) mass spectrometry analysis, we have identified different reaction pathways for the inter-cluster reactions, including partial decomposition-reconstruction and metal exchange reactions, which depends on the structural similarity of the NC reactants involved in the inter-cluster reactions.

## Results and discussion

### Synthesis of parental NCs of [Ag_44_(*p*-MBA)_30_]^4−^ and [Au_44_(*p*-MBA)_26_]^2−^

**Fig. 1:**
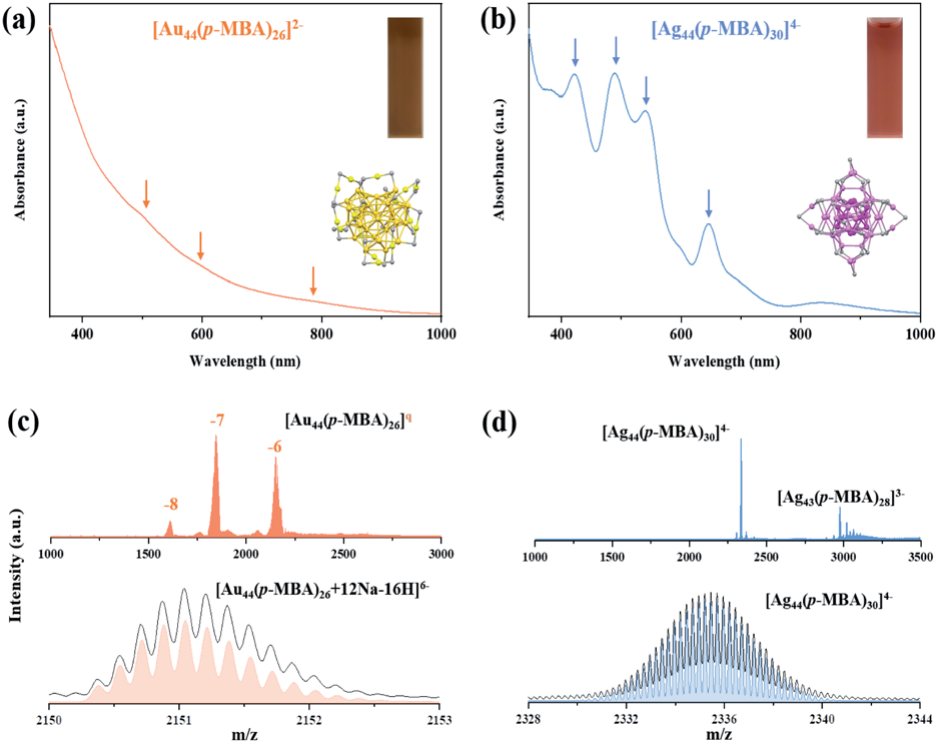
(a and b) UV-vis absorption and (c and d) electrospray ionization (ESI) mass spectra of (a and c) [Au_44_(*p*-MBA)_26_]^2−^ and (b and d) [Ag_44_(*p*-MBA)_30_]^4−^ NCs. Insets in (a and b) show the photographs of *N*,*N*-dimethylformamide (DMF) solution of the corresponding NCs and the crystal structure of [Au_44_(SR)_26_]^2−^ and [Ag_44_(SR)_30_]^4−^ (yellow: Au, purple: Ag, and gray: S; the remaining atoms on the ligand are omitted for clarity). The top panel in (c and d) shows a wide-range ESI mass spectrum, while the bottom spectra show the experimental (black line) and simulated (colored line) isotope patterns of the labeled cluster species.

The parental NCs, [Ag_44_(*p*-MBA)_30_]^4−^ and [Au_44_(*p*-MBA)_26_]^2−^, were synthesized according to reported methods with slight modifications (see the ESI† for more details).^36,43^ The as-prepared [Au_44_(SR)_26_]^2−^ is dark brown in *N*,*N*-dimethylformamide (DMF) solution, and its UV-vis absorption spectrum exhibits peaks at 480, 575, and 765 nm (Fig. 1a). [Ag_44_(SR)_30_]^4−^ is deep red in solution with distinct absorption peaks at 420, 488, 540, 645, and 840 nm (Fig. 1b). The recorded UV-vis absorption spectra of the as-prepared Au_44_ and Ag_44_ NCs are in good agreement with the absorption spectra of pure [Au_44_(*p*-MBA)_26_]^2−^ and [Ag_44_(*p*-MBA)_30_]^4−^, indicating the molecular purity of the as-prepared parental NCs.^36,43^ The high purity and molecular formula of [Ag_44_(SR)_30_]^4−^ and [Au_44_(SR)_26_]^2−^ NCs were further verified by electrospray ionization mass spectrometry (ESI-MS) analysis (Fig. 1c and d). Three sets of peaks were identified at *m*/*z* = 1610, 1843, and 2151 in the wide-range ESI mass spectrum (*m*/*z* = 1000–3000) of [Au_44_(*p*-MBA)_26_]^2−^ (top spectrum, Fig. 1c), which can be assigned to the intact cluster ions carrying 8-, 7-, and 6-charges, respectively. The ESI mass spectrum of [Ag_44_(*p*-MBA)_30_]^4−^ shows the main group of peaks at *m*/*z* = 2335.5 (top spectrum, Fig. 1d), which can be attributed to Ag_44_(*p*-MBA)_30_ NCs carrying 4-charges. It should also be noted that a minor set of peaks was identified at *m*/*z* = 2975, which can be attributed to [Ag_43_(*p*-MBA)_28_]^3−^ (generated by dissociating one unit of Ag(*p*-MBA)_2_ from [Ag_44_(*p*-MBA)_30_]^4−^ during ESI-MS measurement). The cluster and fragmentation peaks recorded in the ESI mass spectra are in good agreement with reported [Ag_44_(*p*-MBA)_30_]^4−^.^35^ The good accuracy of our assignments was further verified by isotope analysis (bottom spectra, Fig. 1c and d), where the respective experimental and simulated isotope patterns of [Au_44_(*p*-MBA)_26_ + 12Na − 16H]^6−^ and [Ag_44_(*p*-MBA)_30_]^4−^ are in perfect agreement. Taken together the UV-vis absorption and ESI mass spectra, we can conclude that atomically precise [Ag_44_(SR)_30_]^4−^ and [Au_44_(SR)_26_]^2−^ NCs have been obtained with molecular purity.

### Inter-cluster reactions at different feeding ratios

To gain insights into the inter-cluster reaction chemistry, [Ag_44_(SR)_30_]^4−^ and [Au_44_(SR)_26_]^2−^ were allowed to react with each other at different feeding ratios (*i.e.*, *R*_Au_44_/Ag_44__ denotes the feeding molar ratio of [Au_44_(SR)_26_]^2−^ to [Ag_44_(SR)_30_]^4−^). Because the stability of [Ag_44_(SR)_30_]^4−^ is highly dependent on the strong coordination between the aprotic solvent molecules and the surface Ag atoms, DMF was chosen as the reaction solvent in this study.^44^ Specifically, [Ag_44_(SR)_30_]^4−^ and [Au_44_(SR)_26_]^2−^ were purified and dispersed in DMF with the same atomic concentration of Au and Ag, and then these two mother solutions were mixed at desired volumetric ratios. The cluster mixture was allowed to stand for another 20 hours to complete the inter-cluster reaction. The final products were then collected without further purification, and they were directly subjected to further characterization. By adjusting the ratio of [Au_44_(SR)_26_]^2−^ and [Ag_44_(SR)_30_]^4−^ (*i.e.*, the value of *R*_Au_44_/Ag_44__) in the reaction, we can obtain Au_*x*_Ag_44−*x*_ NCs with *x* tunable from 1 to 43. In contrast to a constant number of metal atoms (*i.e.*, 44), the number of protecting ligands in the product NCs (*i.e.*, *y* in (Au/Ag)_44_SR_*y*_) can be varied among 26, 27, and 30, which is closely correlated with the structural evolution from [Au_44_(SR)_26_]^2−^ to [Ag_44_(SR)_30_]^4−^.

**Fig. 2:**
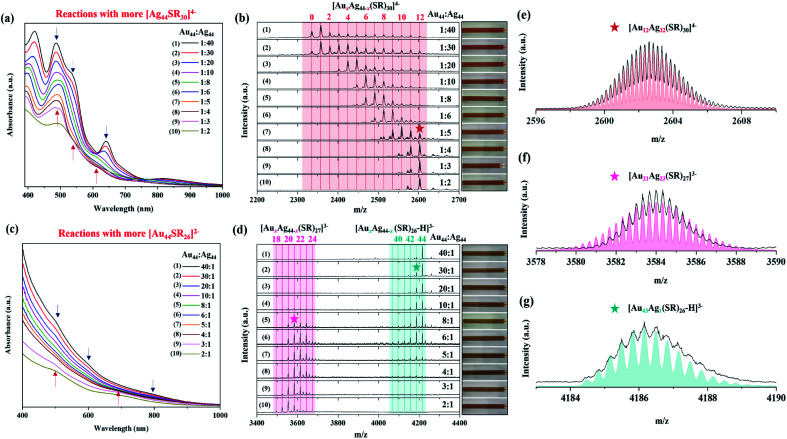
UV-vis absorption (a and c) and ESI mass (b and d) spectra of Au_*x*_Ag_44−*x*_ NCs synthesized using low *R*_Au_44_/Ag_44__ (*i.e.*, 1 : 40 to 1 : 2; more [Ag_44_(SR)_30_]^4−^) (a and b) and high *R*_Au_44_/Ag_44__ (*i.e.*, 2 : 1 to 40 : 1; more [Au_44_(SR)_26_]^2−^) (c and d); (e–g) experimental (black line) and simulated (red, pink, and green lines) mass spectra of [Au_12_Ag_32_(*p*-MBA)_30_]^4−^ (e), [Au_21_Ag_23_(*p*-MBA)_27_]^3−^ (f), and [Au_43_Ag_1_(*p*-MBA)_26_-H]^3−^ (g). Insets of (c and d) show the photographs of the corresponding NC solution. The asterisk indicates the peaks selected from (c) and (d) to illustrate the isotope patterns in (e–f).

One of the most interesting findings is the dosage-dependent M–S framework in the alloy (Au/Ag)_44_ NCs. When more [Ag_44_(SR)_30_]^4−^ NCs (compared to [Au_44_(SR)_26_]^2−^; *R*_Au_44_/Ag_44__ = 1 : 40 to 1 : 2) were added to the reaction mixture, the final NC product of the inter-cluster reaction is [Au_*x*_Ag_44−*x*_(SR)_30_]^4−^ (*x* = 1–12), which preserves the framework of [Ag_44_(SR)_30_]^4−^. As shown in Fig. 2b, the number of Au atoms in [Au_*x*_Ag_44−*x*_(SR)_30_]^4−^ gradually increases from 1 to 12, as the availability of [Au_44_(SR)_26_]^2−^ increases in the reaction (*i.e.*, *R*_Au_44_/Ag_44__ increases from 1 : 40 to 1 : 2). The as-obtained [Au_*x*_Ag_44−*x*_(SR)_30_]^4−^ NCs (*x* = 1–12) exhibit different UV-vis absorption features, and thus they show variable solution color from pink to light brown (Fig. 2a, inset of Fig. 2b). In particular, the dominant species are [Au_1_Ag_43_(SR)_30_]^4−^ and [Ag_44_(SR)_30_]^4−^, when *R*_Au44/Ag44_ is 1/40. In addition to the dominant [Au_1_Ag_43_(SR)_30_]^4−^ and [Ag_44_(SR)_30_]^4−^, a minor amount of [Au_*x*_Ag_44−*x*_(SR)_30_]^4−^ NCs with *x* = 2–12 was also seen in the mass spectrum recorded at *R*_Au_44_/Ag44_ = 1/40 (Fig. 2b, 1st spectrum). The substitution of up to 12 Ag atoms in [Ag_44_(SR)_30_]^4−^ by Au atoms is in good agreement with previous reports,^35,41^ while the *x* value of the dominant species largely depends on the availability of [Au_44_(SR)_26_]^2−^ (*vide infra*). Due to the low *x* values in the dominant alloy NC species at *R*_Au_44_/Ag_44__ = 1/40, the corresponding UV-vis absorption spectra still maintain the same characteristics of [Ag_44_(SR)_30_]^4−^ (Fig. 2a, 1st curve), which suggests that replacing one or few Ag atoms of [Ag_44_(SR)_30_]^4−^ with Au atoms can merely change the electronic structure of the NC products. A facile and effective way to adjust the value of *x* in the dominant [Au_*x*_Ag_44−*x*_(SR)_30_]^4−^ species is to change the dosage of [Au_44_(SR)_26_]^2−^.

By increasing *R*_Au_44_/Ag_44__ while maintaining the same concentration of [Ag_44_(SR)_30_]^4−^, more Au atoms can be incorporated into [Ag_44_(SR)_30_]^4−^. For example, the inter-cluster reaction between [Au_44_(SR)_26_]^2−^ and [Ag_44_(SR)_30_]^4−^ at *R*_Au_44_/Ag_44__ = 1/10 (Fig. 2b, 4th spectrum) produced dominant [Au_*x*_Ag_44−*x*_(SR)_30_]^4−^ NCs with *x* = 6–8. It should be noted that when the dominant *x* increases to 6–8, the adsorption peak at 488 nm in their UV-vis absorption spectra becomes broadened (Fig. 2a, 4th curve). It is possible to further increase the number of Au atoms incorporated in [Au_*x*_Ag_44−*x*_(SR)_30_]^4−^ NCs at a higher feeding dosage of [Au_44_(SR)_26_]^2−^, where the maximal *x* value was recorded to be 12. In particular, [Au_12_Ag_32_(SR)_30_]^4−^ is the dominant species, when *R*_Au_44_/Ag_44__ increases to 1/4, 1/3, and 1/2 (Fig. 2b, 8th, 9th, and 10th spectra), and their UV-vis absorption spectra show typical absorption peaks of [Au_12_Ag_32_(SR)_30_]^4−^ at 390 and 490 nm with three humps at 546, 620, and 728 nm (Fig. 2a, 8th, 9th, and 10th curves; and Fig. S1†). The perfect agreement between the experimental and simulated isotope patterns of [Au_12_Ag_32_(SR)_30_]^4−^ (Fig. 2f) unambiguously verifies that the dominant NC product generated by the inter-cluster reaction is [Au_12_Ag_32_(SR)_30_]^4−^ (the simulated mass spectra for all [Au_*x*_Ag_44−*x*_(SR)_30_]^4−^ NCs (*x* = 1–12) are shown in Fig. S2†). It is worth noting that the UV-vis absorption spectrum of the product NCs obtained at *R*_Au_44_/Ag_44__ = 1/2 is less distinct than those acquired at *R*_Au_44_/Ag_44__ = 1/3 and 1/4 (Fig. 2a, 8th, 9th, and 10th curves). Such diminishing features should be attributed to a small amount of impurities generated in the inter-cluster reaction, as confirmed by ESI-MS analysis (Fig. S3†). As the feeding atomic ratio of Au/Ag at *R*_Au_44_/Ag_44__ = 1/2 is larger than the Au/Ag ratio in [Au_12_Ag_32_(SR)_30_]^4−^ (1/2.6), there should be an excess of [Au_44_(SR)_26_]^2−^ in the reaction solution. However, we did not identify any [Au_44_(SR)_26_]^2−^ in the mass spectrum of the final product at *R*_Au_44_/Ag_44__ = 1/2, where extensive signals corresponding to Au/Ag-SR complexes were observed. Therefore, we speculate that partial decomposition of the two parental NCs occurred as a side reaction accompanying the inter-cluster reaction.

Interestingly, when [Au_44_(SR)_26_]^2−^ dominates the reaction mixture, the reaction between [Ag_44_(SR)_30_]^4−^ and [Au_44_(SR)_26_]^2−^ tends to form [Au_*x*_Ag_44−*x*_(SR)_26_]^2−^ NCs (*i.e.*, the same ligand number of 26 as [Au_44_(SR)_26_]^2−^), which adopt the same M–S framework as [Au_44_(SR)_26_]^2−^. As shown in the top three spectra in Fig. 2d, the introduction of a small amount of [Ag_44_(SR)_30_]^4−^ (*R*_Au_44_/Ag_44__ = 40/1, 30/1, and 20/1) in the reaction produced Au-rich alloy NCs (*i.e.*, [Au_*x*_Ag_44−*x*_(SR)_26_]^2−^ with *x* = 42–44). The perfect match between the experimental and simulated isotope patterns of [Au_43_Ag_1_(*p*-MBA)_26_-H]^3−^ and [Au_42_Ag_2_(*p*-MBA)_26_-H]^3−^ confirms the accuracy of our mass spectrum assignment (Fig. 2g, S4a and b†). Similar to [Au_*x*_Ag_44−*x*_(SR)_30_]^4−^ with *x* = 0–1 (Fig. 2a, 1st curve), slight Ag doping also has a marginal effect on the electronic structure of [Au_44_(SR)_26_]^2−^, which is evidenced by its almost identical UV-vis absorption spectrum to that of [Au_*x*_Ag_44−*x*_(SR)_26_]^2−^ (*x* = 42–44; Fig. 2c, 1st, 2nd, and 3rd curves). More interestingly, as the *R*_Au_44_/Ag_44__ ratio decreases to 10/1, 8/1, and 6/1, except for the Au-rich [Au_*x*_Ag_44−*x*_(SR)_26_]^2−^ NCs (*x* = 39–43) with decreasing number of Au atoms, a set of new peaks at *m*/*z* = 3500–3700 were observed in the mass spectra of the product NCs (Fig. 2d, 4th–6th spectra). These new cluster peaks can be assigned to [Au_*x*_Ag_44−*x*_(SR)_27_]^3−^ (*x* = 20–24), with a new ligand number of 27 and a comparable number of Ag and Au atoms. The accuracy of our assignment was verified by the isotope pattern analysis of [Au_21_Ag_23_(SR)_27_]^3−^, where the experimental and simulated patterns are in good agreement (Fig. 2f; the simulated mass spectra of [Au_*x*_Ag_44−*x*_(SR)_27_]^3−^ NCs (*x* = 20–22) are shown in Fig. S5a–d†). Since these two sets of alloy NCs ([Au_*x*_Ag_44−*x*_(SR)_27_]^3−^ and [Au_*x*_Ag_44−*x*_(SR)_26_]^2−^) coexist in the product solution, the UV-vis absorption spectra exhibit less distinct peaks (Fig. 2c, 4th–6th curves). As the ratio of [Ag_44_(SR)_30_]^4−^ (*R*_Au_44_/Ag_44__ = 5/1 and 4/1) in the reaction further increases, the peak intensity of [Au_*x*_Ag_44−*x*_(SR)_26_]^2−^ decreases, while those of [Au_*x*_Ag_44−*x*_(SR)_27_]^3−^ increase (Fig. 2d, 7th and 8th spectra). Based on the above observation, it is obvious that a structural transformation from [Au_*x*_Ag_44−*x*_(SR)_26_]^2−^ into [Au_*x*_Ag_44−*x*_(SR)_27_]^3−^ would occur as the Ag content in the framework of Au_*x*_Ag_44−*x*_ increases. The Ag content-induced structural transformation was further verified by the increased abundance of [Au_*x*_Ag_44−*x*_(SR)_27_]^3−^ with the increasing dosage of [Ag_44_(SR)_30_]^4−^. In particular, [Au_*x*_Ag_44−*x*_(SR)_27_]^3−^ becomes the dominant product at *R*_Au_44_/Ag_44__ = 3/1 and 2/1 (Fig. 2d, 9th and 10th spectra). Although the same alloy framework of [Au_*x*_Ag_44−*x*_(SR)_27_]^3−^ can be obtained at *R*_Au_44_/Ag_44__ = 3/1 and 2/1, the specific content of Ag seems to be tunable by the feeding ratio of [Ag_44_(SR)_30_]^4−^. The dominant *x* values were recorded to be 19–21 at *R*_Au_44_/Ag_44__ = 2/1, while those documented at *R*_Au_44_/Ag_44__ = 3/1 were 20–22 (Fig. 2d, 9th and 10th spectra, Fig. S5†). Taken together, by changing the dosage of [Ag_44_(SR)_30_]^4−^ while keeping [Au_44_(SR)_26_]^2−^ as the dominant cluster (*i.e.*, *R*_Au_44_/Ag_44__ = 2 : 1 to 40 : 1) in the feeding cluster mixture, two families of alloy NCs with different M–S frameworks can be produced, including [Au_*x*_Ag_44−*x*_(SR)_26_]^2−^ (*x* = 40–43) and [Au_*x*_Ag_44−*x*_(SR)_27_]^3−^ (*x* = 19–24), where the former features rich Au atoms and the latter possesses comparable Au and Ag atoms.

In general, the Au/Ag ratio in the final alloy NC products decreases with the decrease of feeding *R*_Au_44_/Ag_44__ (Fig. S6†). In particular, only a few Ag atoms can be incorporated into [Au_*x*_Ag_44−*x*_(SR)_26_]^2−^ (average Au/Ag ratio >80) under high feeding *R*_Au_44_/Ag_44__ (10 < *R*_Au_44_/Ag_44__ < 40) (Fig. S6a†). With the decrease of *R*_Au_44_/Ag_44__ (0.5 < *R*_Au_44_/Ag_44__ < 8), the alloy frameworks change to [Au_*x*_Ag_44−*x*_(SR)_27_]^3−^ with an average Au/Ag ratio of 0.4 to 3.7 (Fig. S6b†). Further decreasing the *R*_Au_44_/Ag_44__ to below 0.5, the alloy products can keep the framework of [Au_*x*_Ag_44−*x*_(SR)_30_]^4−^ with an average Au/Ag ratio ranging from 0.1 to 0.4 (Fig. S6c†). In addition to the metal ratios and structures, the valence electron count of the alloy NC products is also highly related to those of the reactants. In particular, the valence electron count of [Au_44_(SR)_26_]^2−^ is 20, while that of [Ag_44_(SR)_30_]^4−^ is 18. Therefore, tuning the *R*_Au_44_/Ag_44__ would also change the number of electrons participating in the reactions. As shown in Table S1,† when the average feeding electrons are less than 19, the alloy products are [Au_*x*_Ag_44−*x*_(SR)_30_]^4−^ NCs featuring a valence electron count of 18. If the average feeding electrons increase to more than 19, the inter-cluster reactions produce [Au_*x*_Ag_44−*x*_(SR)_26_]^2−^ and [Au_*x*_Ag_44−*x*_(SR)_27_]^3−^ NCs, whose valence electron counts are 20.

### Real-time monitoring of the inter-cluster reactions

To the best of our knowledge, Au_*x*_Ag_44−*x*_ NCs protected by 27 thiolate ligands have not been observed in previous reports, and [Au_*x*_Ag_44−*x*_(SR)_27_]^3−^ represents a structural transition between Au-rich [Au_*x*_Ag_44−*x*_(SR)_26_]^2−^ and Ag-rich [Au_*x*_Ag_44−*x*_(SR)_30_]^4−^ NCs. In order to study the inter-cluster reaction between [Au_44_(SR)_26_]^2−^ and [Ag_44_(SR)_30_]^4−^, as well as the structural evolution of the alloy Au_*x*_Ag_44−*x*_ NCs, we conducted real-time UV-vis absorption spectroscopy and ESI-MS analysis of three representative reactions at *R*_Au_44_/Ag_44__ = 1/3, 3/1, and 6/1, where [Au_12_Ag_32_(SR)_30_]^4−^, [Au_*x*_Ag_44−*x*_(SR)_27_]^3−^ (*x* = 20–22), and [Au_*x*_Ag_44−*x*_(SR)_26_]^2−^ (*x* = 42–44) were obtained as prominent products, respectively.

**Fig. 3:**
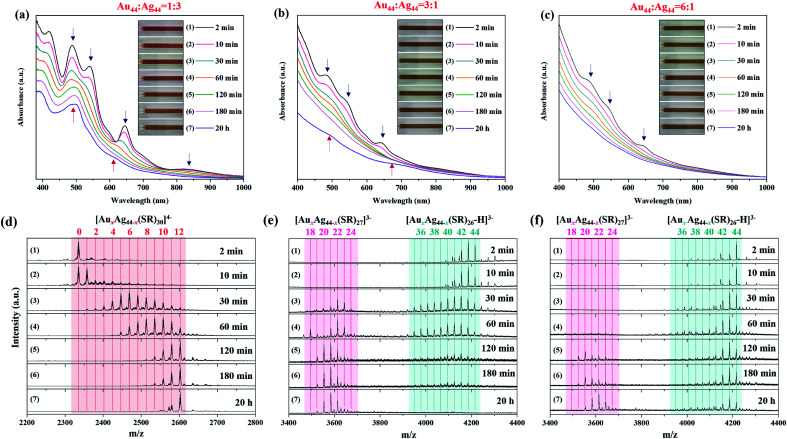
Time-course UV-vis absorption spectra of the inter-cluster reaction between [Au_44_(SR)_26_]^2−^ and [Ag_44_(SR)_30_]^4−^ at a *R*_Au44/Ag44_ of (a) 1 : 3, (b) 3 : 1, and (c) 6 : 1. Time-course ESI mass spectra of the inter-cluster reaction between [Au_44_(SR)_26_]^2−^ and [Ag_44_(SR)_30_]^4−^ at a *R*_Au44/Ag44_ of (d) 1 : 3, (e) 3 : 1, and (f) 6 : 1. The insets in (a–c) show the digital images of the corresponding reaction solutions taken at different reaction times.

In the reaction system of *R*_Au44/Ag44_ = 1/3, real-time UV-vis absorption and ESI mass spectra show that [Ag_44_(SR)_30_]^4−^ gradually evolves into [Au_12_Ag_32_(SR)_30_]^4−^, and the number of Au atoms gradually increases in [Au_*x*_Ag_44−*x*_(SR)_30_]^4−^ (Fig. 3a and d). Specifically, at the initial reaction time (*t* = 2 min), [Ag_44_(SR)_30_]^4−^ is still the main species in the reaction mixture with a negligible amount of [Au_1_Ag_43_(SR)_30_]^4−^ (Fig. 3d, 1st spectrum). However, regarding the Au-related species, only a few new peaks of Au-SR complexes appear at *m*/*z* = 1000–2000 (Fig. S7†), indicating that [Au_44_(SR)_26_]^2−^ has been decomposed after mixing with [Ag_44_(SR)_30_]^4−^. As the reaction proceeds to *t* = 30 and 60 min, the dominant species have developed into [Au_*x*_Ag_44−*x*_(SR)_30_]^4−^ (*x* > 5), and the increase of Au doping induces a significant change in the UV-vis absorption spectra, where the absorption peak at 488 nm broadens and the peak intensity at 646 nm deceases sharply (Fig. 3a, 5th and 6th curves, and Fig. 3d, 3rd and 4th spectra). As the reaction time increases to *t* = 120 and 180 min, the population of [Au_*x*_Ag_44−*x*_(SR)_30_]^4−^ shifts toward the high *x* end, where the maximal *x* value is 12 (Fig. 3d, 5th and 6th spectra). More interestingly, as this reaction proceeds to 20 h, [Au_12_Ag_32_(SR)_30_]^4−^ gradually becomes dominant in the final product *via* a size-focusing process (Fig. 3a, 7th curve and Fig. 3d, 7th spectrum). It should be noted that the atomic ratio of Au/Ag in [Au_12_Ag_32_(SR)_30_]^4−^ and [Au_11_Ag_33_(SR)_30_]^4−^ is 1/2.6 and 1/3, respectively, which is close to the feeding ratio of Au_44_/Ag_44_ = 1/3. These data also suggest that the atomic efficiency of the inter-cluster reaction is high. Therefore, in the presence of excess [Ag_44_(SR)_30_]^4−^, [Au_44_(SR)_26_]^2−^ will react with [Ag_44_(SR)_30_]^4−^ most likely through the decomposition mechanism, where [Au_44_(SR)_26_]^2−^ will decompose into smaller cluster or complex species. The as-formed smaller cluster or complex species will then react with [Ag_44_(SR)_30_]^4−^, resulting in the substitution of Ag atoms by Au atoms while keeping the M–S framework of [Ag_44_(SR)_30_]^4−^ unchanged. With sufficient supply of [Au_44_(SR)_26_]^2−^, this decomposition-substitution mechanism can incorporate up to 12 Au heteroatoms into [Au_*x*_Ag_44−*x*_(SR)_30_]^4−^ NCs.

On the other hand, when the inter-cluster reaction starts with an excess of [Au_44_(SR)_26_]^2−^ NCs, the structural evolution pathway is distinctly different from the above one. Taking the reaction at *R*_Au_44_/Ag_44__ = 3/1 as an example, there are several Ag atoms incorporated into the framework of [Au_44_(SR)_26_]^2−^, leading to the formation of [Au_*x*_Ag_44−*x*_(SR)_26_]^2−^ NCs with *x* = 40–43 in the initial stage (*t* = 2 and 10 min; Fig. 3e, 1st and 2nd spectra). Meanwhile, ESI mass spectra in a broad *m*/*z* range show a set of peaks at *m*/*z* = 2200–2600, which can be attributed to [Au_*x*_Ag_44−*x*_(L)_30_]^4−^ (*x* = 1–12; L denotes SR or Cl) (Fig. S8†). It should be noted that up to 3 Cl ligands can be incorporated into the protecting shell of [Au_*x*_Ag_44−*x*_(L)_30_]^4−^ (*x* = 1–12), which suggests that the alloying reaction is most probably initiated by disturbing the protecting shell of [Ag_44_(SR)_30_]^4−^. [Au_*x*_Ag_44−*x*_(L)_30_]^4−^ NCs (*x* = 1–12) NCs were consumed in the next stage of reaction (*t* = 30 and 60 min), accompanied by an increase of Ag doping in [Au_*x*_Ag_44−*x*_(SR)_26_]^2−^ NCs with *x* = 35–43 (Fig. 3e, 3rd and 4th spectra). In the meantime, with the diminishing of [Au_*x*_Ag_44−*x*_(L)_30_]^4−^ (*x* = 1–12) there is the emergence of [Au_*x*_Ag_44−*x*_(SR)_27_]^3−^ NCs with *x* = 17–24 (Fig. 3e, 3rd and 4th spectra). As the reaction proceeds to *t* = 120 and 180 min, more [Au_*x*_Ag_44−*x*_(SR)_27_]^3−^ NCs were formed at the expense of [Au_*x*_Ag_44−*x*_(SR)_26_]^2−^ (Fig. 3e, 5th and 6th spectra). The UV-vis absorption spectra taken at this stage (*t* = 120 and 180 min) show no distinct peaks, which are most probably the miscellaneous and partially overlapping absorption features of [Au_*x*_Ag_44−*x*_(SR)_26_]^2−^ and [Au_*x*_Ag_44−*x*_(SR)_27_]^3−^ (Fig. 3b, 5th and 6th curves). At the last stage of the reaction (*t* = 20 h), [Au_*x*_Ag_44−*x*_(SR)_27_]^3−^ becomes dominant, and its *x* distribution is narrowed to *x* = 20–22, while [Au_*x*_Ag_44−*x*_(SR)_26_]^2−^ has extinguished. Therefore, the two shoulder peaks at 500 and 690 nm in the 7th curve of Fig. 3b should be attributed to [Au_*x*_Ag_44−*x*_(SR)_27_]^3−^ (*x* = 20–22). To the best of our knowledge, this is the first report on the production of [Au_*x*_Ag_44−*x*_(SR)_27_]^3−^ NCs with molecular purity and a well-defined absorption spectrum.

To understand the structural correlation between [Au_*x*_Ag_44−*x*_(SR)_27_]^3−^ (*x* = 19–24) and Au-rich [Au_*x*_Ag_44−*x*_(SR)_26_]^2−^ (*x* = 40–43) NCs, we performed the inter-cluster reaction at a higher dosage of [Au_44_(SR)_26_]^2−^. The reaction at *R*_Au_44_/Ag_44__ = 6/1 produced a composition and structure evolution pathway similar to that at *R*_Au_44_/Ag_44__ = 3/1 (Fig. 3c, f and S9†). Specifically, Ag atoms are first doped into [Au_44_(SR)_26_]^2−^ to produce [Au_*x*_Ag_44−*x*_(SR)_26_]^2−^ (*x* = 40–43, Fig. 3f, 1st and 2nd spectra). As the amount of Ag doping increases, [Au_*x*_Ag_44−*x*_(SR)_27_]^3−^ appears and becomes more abundant (Fig. 3f, 5th–7th spectrum). Since more [Au_44_(SR)_26_]^2−^ NCs are available at *R*_Au_44_/Ag_44__ = 6/1 than that at *R*_Au_44_/Ag_44__ = 3/1, it took a longer time (∼120 min) for [Au_*x*_Ag_44−*x*_(SR)_27_]^3−^ to be consumed (Fig. 3f, 5th spectrum). The final product obtained at *R*_Au_44_/Ag_44__ = 6/1 is a mixture of [Au_*x*_Ag_44−*x*_(SR)_26_]^2−^ and [Au_*x*_Ag_44−*x*_(SR)_27_]^3−^, which means that the limited availability of [Ag_44_(SR)_30_]^4−^ in the reaction solution is not able to convert all Au-rich [Au_*x*_Ag_44−*x*_(SR)_26_]^2−^ into [Au_*x*_Ag_44−*x*_(SR)_27_]^3−^ (Fig. 3f, 7th spectrum). Similarly, the co-existence of [Au_*x*_Ag_44−*x*_(SR)_26_]^2−^ and [Au_*x*_Ag_44−*x*_(SR)_27_]^3−^ produces an apparently featureless UV-vis absorption spectrum (Fig. 3c, 7th curve).

Based on the above observation, we are now able to propose a plausible mechanism for the inter-cluster and alloying reaction between [Ag_44_(SR)_30_]^4−^ and [Au_44_(SR)_26_]^2−^. The most important finding in the inter-cluster reaction is the feeding dosage-dependent M–S framework in the final alloy NCs, which stems from the composition-sensitive stability of the M–S framework. Taking the inter-cluster reaction in the presence of excess [Au_44_(SR)_26_]^2−^ NCs as an example, the reaction between [Au_44_(SR)_26_]^2−^ and [Ag_44_(SR)_30_]^4−^ would first induce metal exchange between two parental NCs, giving rise to [Au_*x*_Ag_44−*x*_(SR)_26_]^2−^ and [Au_*x*_Ag_44−*x*_(SR)_30_]^4−^. Due to the excess availability of [Au_44_(SR)_26_]^2−^, the increasing Au doping in [Au_*x*_Ag_44−*x*_(SR)_30_]^4−^ will sharply disturb its structural stability, thereby inducing its decomposition in the early stage of the reaction (*e.g.*, *t* = 2–10 min at *R*_Au_44_/Ag_44__ = 3/1). The decomposed Ag-rich [Au_*x*_Ag_44−*x*_(SR)_30_]^4−^ will then serve as an Ag source to react with Au-rich [Au_*x*_Ag_44−*x*_(SR)_26_]^2−^, increasing the doping amount of Ag in the latter. On the other hand, as the amount of Ag doping in [Au_*x*_Ag_44−*x*_(SR)_26_]^2−^ increases, the structural stability of M_44_S_26_ will be compromised, which induces the transformation of Au-rich [Au_*x*_Ag_44−*x*_(SR)_26_]^2−^ into [Au_*x*_Ag_44−*x*_(SR)_27_]^3−^. Therefore, the composition and the M–S framework of the final NC products are determined by the feeding ratio of [Au_44_(SR)_26_]^2−^ and [Ag_44_(SR)_30_]^4−^. In general, the feeding ratio of [Au_44_(SR)_26_]^2−^ and [Ag_44_(SR)_30_]^4−^ determines the composition of the metal core in (Au/Ag)_44_ NCs, and the composition of the metal core will further direct the formation of the M–S framework. The Au-rich metal core prefers the M_44_S_26_ framework of [Au_*x*_Ag_44−*x*_(SR)_26_]^2−^ (*x* = 40–43), while the Ag-rich core favors the M_44_S_30_ framework of [Au_*x*_Ag_44−*x*_(SR)_30_]^4−^ (*x* = 1–12). An evenly mixed Au/Ag core would lead to the formation of a M_44_S_27_ framework in [Au_*x*_Ag_44−*x*_(SR)_27_]^3−^ (*x* = 19–24). On the other hand, as the two parental NCs are both capped by *p*-MBA ligands, it is also crucial to investigate how the ligands involve in the actual exchange of metal atoms. We performed the inter-cluster reaction between [Au_44_(*p*-MBA)_26_]^2−^ and bi-ligand protected [Ag_44_(*p*-MBA)_30−*y*_(NTP)_*y*_]^4−^ NCs (*y* = 0, 3, 5, 9) (Fig. S10†) with different *R*_Au_44_/Ag_44__. As shown in Fig. S11,† the final alloy products synthesized from bi-ligand protected [Ag_44_(*p*-MBA)_30−*y*_(NTP)_*y*_]^4−^ NCs show consistent UV-vis absorption features with those synthesized by mono-ligand protected [Ag_44_(*p*-MBA)_30_]^4−^ NCs, indicating their similar metal composition and structure. Therefore, in this inter-cluster reaction, the ligands show marginal effects on the reaction fate, as long as the ligands are capable of maintaining the geometric structure of the parental NCs. A similar observation has been reported in inter-cluster reaction literature, where the heterogeneity in the ligand shell can accelerate the inter-cluster reaction kinetics, but hardly affects the profile of final products.^28^

### Transformation between different sets of (Au/Ag)_44_ NCs

**Fig. 4:**
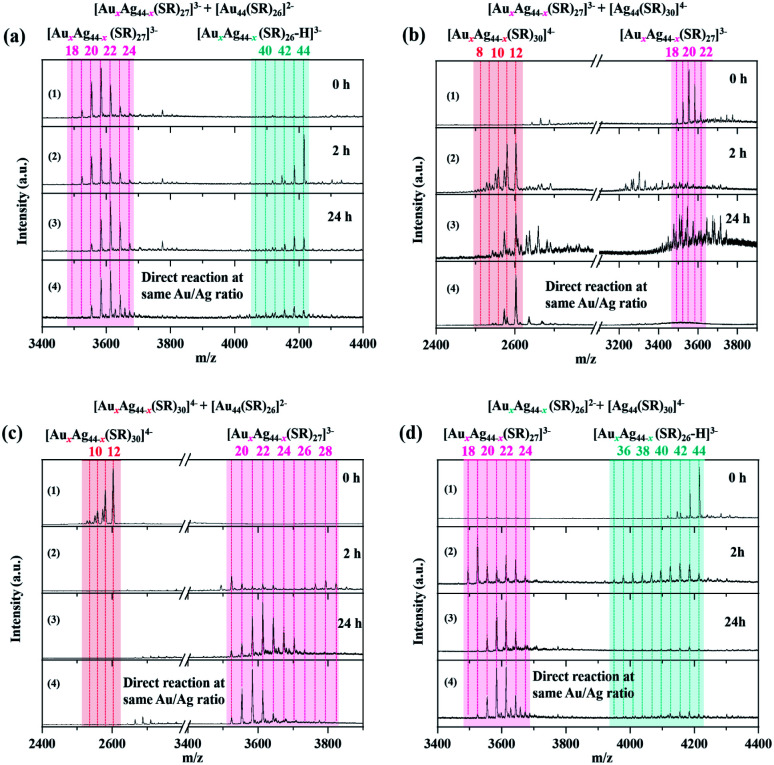
(a) ESI mass spectra of the reactions between [Au_*x*_Ag_44−*x*_(SR)_27_]^3−^ and [Au_44_(SR)_26_]^2−^ at the reaction times of *t* = 2 and 24 h (*R*_Au_44_/Ag_44__ = 5/1). (b) ESI mass spectra of the reactions between [Au_*x*_Ag_44−*x*_(SR)_27_]^3−^ and [Ag_44_(SR)_30_]^4−^ at the reaction times of *t* = 2 and 24 h (*R*_Au_44_/Ag_44__ = 1/2). (c) ESI mass spectra of the reactions between [Au_*x*_Ag_44−*x*_(SR)_30_]^4−^ and [Au_44_(SR)_26_]^2−^ at the reaction times of *t* = 2 and 24 h (*R*_Au_44_/Ag_44__ = 3/1). (d) ESI mass spectra of the reactions between [Au_*x*_Ag_44−*x*_(SR)_26_]^2−^ and [Ag_44_(SR)_30_]^4−^ at the reaction times of *t* = 2 and 24 h (*R*_Au_44_/Ag_44__ = 4/1). The bottom spectrum of each panel is the ESI mass spectrum of the alloy NCs produced by the direct reaction of [Au_44_(SR)_26_]^2−^ and [Ag_44_(SR)_30_]^4−^, where *R*_Au_44_/Ag_44__ is equal to the above reactions.

In order to study the relationship among the three sets of alloy NCs produced by the inter-cluster reaction between [Au_44_(SR)_26_]^2−^ and [Ag_44_(SR)_30_]^4−^, the as-obtained Au-rich [Au_*x*_Ag_44−*x*_(SR)_26_]^2−^, evenly mixed [Au_*x*_Ag_44−*x*_(SR)_27_]^3−^, and Ag-rich [Au_*x*_Ag_44−*x*_(SR)_30_]^4−^ NCs were used to further react with [Au_44_(SR)_26_]^2−^ or [Ag_44_(SR)_30_]^4−^. We first focus on the cluster reaction of [Au_*x*_Ag_44−*x*_(SR)_27_]^3−^, which was prepared by the inter-cluster reaction between [Ag_44_(SR)_30_]^4−^ and [Au_44_(SR)_26_]^2−^ at *R*_Au_44_/Ag_44__ = 3/1 (1st spectrum of Fig. 4a and b). The as-prepared [Au_*x*_Ag_44−*x*_(SR)_27_]^3−^ NCs were further reacted with [Au_44_(SR)_26_]^2−^ and [Ag_44_(SR)_30_]^4−^. As can be seen in Fig. 4a and b, the reaction of [Au_*x*_Ag_44−*x*_(SR)_27_]^3−^ with [Au_44_(SR)_26_]^2−^ and [Ag_44_(SR)_30_]^4−^ adopts different reaction pathways, leading to the formation of different NC products. Upon mixing [Au_44_(SR)_26_]^2−^ (two equivalents to [Ag_44_(SR)_30_]^4−^ used to produce [Au_*x*_Ag_44−*x*_(SR)_27_]^3−^) with [Au_*x*_Ag_44−*x*_(SR)_27_]^3−^, these two sets of NCs coexist in the reaction mixture, and a few Ag heteroatoms can be incorporated into [Au_44_(SR)_26_]^2−^ within 2 h, leading to the formation of [Au_*x*_Ag_44−*x*_(SR)_26_]^2−^ (*x* = 42–43; Fig. 4a, 2nd spectrum). It should be noted that since [Au_*x*_Ag_44−*x*_(SR)_27_]^3−^ was prepared at *R*_Au_44_/Ag_44__ = 3/1, adding additional two equivalents of [Au_44_(SR)_26_]^2−^ (relative to [Ag_44_(SR)_30_]^4−^) makes a total ratio of *R*_Au_44_/Ag_44__ = 5/1 in the reaction mixture. In addition to the early stage of the reaction, we also monitored the products of the inter-cluster reaction at the steady stage (*i.e.*, *t* = 24 h). Compared with the ESI mass spectra in the early stage of the cluster reaction (Fig. 4a, 2nd spectrum), the most notable change in the ESI mass spectrum at *t* = 24 h is that the peak intensity of [Au_44_(SR)_26_]^2−^ is significantly deceased, accompanied by an increase of the maximal *x* in [Au_*x*_Ag_44−*x*_(SR)_27_]^3−^. These data suggest that the Au content in [Au_*x*_Ag_44−*x*_(SR)_27_]^3−^ is increased at the expense of [Au_44_(SR)_26_]^2−^. It is worth noting that the ESI mass spectrum of the final product is almost identical to that of the alloy NCs produced by directly mixing [Au_44_(SR)_26_]^2−^ and [Ag_44_(SR)_30_]^4−^ at the same Au/Ag ratio (*i.e.*, *R*_Au_44_/Ag_44__ = 5/1; Fig. 4a, 4th spectrum). The above observation also indicates that the introduction of [Au_44_(SR)_26_]^2−^ into [Au_*x*_Ag_44−*x*_(SR)_27_]^3−^ cannot change the M–S framework of parental NCs, but it can induce metal exchange between the parental NCs, generating alloy NCs that are identical to those produced by the direct reaction of [Au_44_(SR)_26_]^2−^ and [Ag_44_(SR)_30_]^4−^. Therefore, we speculate that the structure of newly discovered [Au_*x*_Ag_44−*x*_(SR)_27_]^3−^ is close to that of [Au_44_(SR)_26_]^2−^. Taking the moderate number of SR ligands bridging those of [Ag_44_(SR)_30_]^4−^ and [Au_44_(SR)_26_]^2−^ into consideration, [Au_*x*_Ag_44−*x*_(SR)_27_]^3−^ is most probably formed by hetero-dimerization of [Ag_44_(SR)_30_]^4−^ and [Au_44_(SR)_26_]^2−^, similar to the recently discovered hetero-dimeric structure of Au_29_(SR)_19_ NCs composed of a half structure of Au_30_(SR)_18_ and a half structure of Au_28_(SR)_20_.^45^ It should be pointed out that the alloying reactions made possible by the apparent metal exchange between NCs with similar or identical structures (*e.g.*, Ag_25_SR_18_/Au_25_SR_18_, and Au_38_(SR)_24_/Ag_*x*_Au_38−*x*_(SR)_24_) have been well demonstrated in the literature.^24,46^

In order to provide more information about the structural similarity among the (Au/Ag)_44_ alloy NCs, we compared the ion mobility of [Au_44_(SR)_26_]^2−^, [Au_*x*_Ag_44−*x*_(SR)_26_]^2−^, [Au_*x*_Ag_44−*x*_(SR)_27_]^3−^, and [Au_*x*_Ag_44−*x*_(SR)_30_]^4−^ NCs in polyacrylamide gel electrophoresis (PAGE). As shown in Fig. S12,† with the increase of the Ag atom ratio in the alloy (Au/Ag)_44_ frameworks, the mobility of alloy NCs increases due to the larger charge-to-mass ratio. More importantly, the mobility of [Au_*x*_Ag_44−*x*_(SR)_27_]^3−^ falls in between those of [Au_*x*_Ag_44−*x*_(SR)_26_]^2−^ and [Au_*x*_Ag_44−*x*_(SR)_30_]^4−^, indicating that [Au_*x*_Ag_44−*x*_(SR)_27_]^3−^ NCs most probably share a combined structural feature of [Au_44_(SR)_26_]^2−^ and [Ag_44_(SR)_30_]^4−^ NCs. In addition, tandem mass (MS/MS) analysis was also conducted to compare the fragmentation behavior of [Au_*x*_Ag_44−*x*_(SR)_27_]^3−^ and [Au_12_Ag_32_(SR)_30_]^4−^ NCs, which can further provide information on the surface features of targeted alloy NCs (Fig. S13 and S14†). In particular, the 4-peak of [Au_12_Ag_32_(SR)_30_]^4−^ was chosen as a parent ion in the MS/MS analysis. As shown in Fig. S13,† [Au_12_Ag_32_(SR)_30_]^4−^ can be fragmented in a stripping-off way during the MS/MS process, where the fragment cluster ions were produced by successive dissociation of [M(SR)_2_]^−^ (M = Au and Ag). A similar preferential dissociation of [Au(SR)_2_]^−^ was observed in the MS/MS spectra of [Au_21_Ag_23_(SR)_27_-H]^4−^. As shown in Fig. S14,† as the collision energy increases from 5 to 20 eV, [Au(SR)_2_]^−^ was dissociated from [Au_21_Ag_23_(SR)_27_-H]^4−^ to generate [Au_20_Ag_23_(SR)_25_-H]^3−^. A similar dissociation of [M(SR)_2_]^−^ in their fragmentation process suggests structural similarity between [Au_*x*_Ag_44−*x*_(SR)_27_]^3−^ and [Au_*x*_Ag_44−*x*_(SR)_30_]^4−^, further supporting the above PAGE data.

By contrast, the reaction between [Ag_44_(SR)_30_]^4−^ and [Au_*x*_Ag_44−*x*_(SR)_27_]^3−^ proceeds through a completely different mechanism. [Au_*x*_Ag_44−*x*_(SR)_27_]^3−^ was prepared by an inter-cluster reaction between [Ag_44_(SR)_30_]^4−^ and [Au_44_(SR)_26_]^2−^ at *R*_Au_44_/Ag_44__ = 2/1, followed by adding another 3 equivalents of [Ag_44_(SR)_30_]^4−^ into the as-prepared [Au_*x*_Ag_44−*x*_(SR)_27_]^3−^ to make the total *R*_Au_44_/Ag_44__ = 1/2. One of the most noticeable differences is the accelerated reaction kinetics between [Ag_44_(SR)_30_]^4−^ and [Au_*x*_Ag_44−*x*_(SR)_27_]^3−^, which is readily indicated by the diminishing of [Au_*x*_Ag_44−*x*_(SR)_27_]^3−^ in their ESI mass spectrum after 2 h of mixing (Fig. 4b, 2nd spectrum). In addition, we also captured a larger number of Au-SR, Ag-SR, and Ag–Au-SR complexes in the reaction system of [Au_*x*_Ag_44−*x*_(SR)_27_]^3−^ and [Ag_44_(SR)_30_]^4−^, in comparison to that observed in its reaction with [Au_44_(SR)_26_]^2−^ (Fig. S15†). The extensive formation of Au/Ag-SR complexes should be attributed to the decomposition of the parental [Au_*x*_Ag_44−*x*_(SR)_27_]^3−^ and [Ag_44_(SR)_30_]^4−^ NCs. The composition of the final NC product still depends on the dosage of feeding NCs. As the total *R*_Au_44_/Ag_44__ is 1/2, the inter-cluster reaction between [Au_*x*_Ag_44−*x*_(SR)_27_]^3−^ and [Ag_44_(SR)_30_]^4−^ produced [Au_12_Ag_32_(SR)_30−*b*_Cl_*b*_]^4−^ (*b* = 0–1) as the main product. Besides the prominent peaks of [Au_12_Ag_32_(SR)_30−*b*_Cl_*b*_]^4−^ (*b* = 0–1), several peaks can also be identified at *m*/*z* = 3200–3800 (Fig. 4b, 2nd and 3rd spectra), which should be the side products from the decomposition of [Au_*x*_Ag_44−*x*_(SR)_27_]^3−^. However, their detailed assignment is impossible due to the low signal-to-noise ratio. Of particular note, the mass spectrum pattern of the final product delivered by the inter-cluster reaction between [Au_*x*_Ag_44−*x*_(SR)_27_]^3−^ and [Ag_44_(SR)_30_]^4−^ is inconsistent with that produced by the direct reaction of [Au_44_(SR)_26_]^2−^ and [Ag_44_(SR)_30_]^4−^ (Fig. 4b, 3rd and 4th spectra). These data suggest that the alloying reaction between [Ag_44_(SR)_30_]^4−^ and [Au_*x*_Ag_44−*x*_(SR)_27_]^3−^ might occur through a decomposition-reconstruction mechanism, where the parental NCs would decompose into smaller NC or complex species. The re-growth or combination of such smaller NC or complex species would lead to the formation of the final NC product, [Au_12_Ag_32_(SR)_30−*b*_Cl_*b*_]^4−^ (*b* = 0–1).

More interestingly, [Au_*x*_Ag_44−*x*_(SR)_26_]^2−^ and [Au_*x*_Ag_44−*x*_(SR)_30_]^4−^ can also transform into [Au_*x*_Ag_44−*x*_(SR)_27_]^3−^ by the inter-cluster reaction with [Ag_44_(SR)_30_]^4−^ and [Au_44_(SR)_26_]^2−^, respectively. As shown in Fig. 4c, by further introducing [Au_44_(SR)_26_]^2−^ into [Au_*x*_Ag_44−*x*_(SR)_30_]^4−^ (*x* = 10–12; produced by the inter-cluster reaction of [Au_44_(SR)_26_]^2−^ and [Ag_44_(SR)_30_]^4−^ at *R*_Au_44_/Ag_44__ = 1/3) and changing the *R*_Au_44_/Ag_44__ to 3 : 1, the peaks assigned to [Au_*x*_Ag_44−*x*_(SR)_30_]^4−^ disappear in the mass spectra. Together with the disappearance of [Au_*x*_Ag_44−*x*_(SR)_30_]^4−^ is the increasing abundance of [Au_*x*_Ag_44−*x*_(SR)_27_]^3−^, where the range of *x* is narrowed down from 18–29 to 19–23 (Fig. 4c, 2nd and 3rd spectra). In addition, due to the notable structural differences between [Au_*x*_Ag_44−*x*_(SR)_30_]^4−^ and [Au_*x*_Ag_44−*x*_(SR)_27_]^3−^, several Au/Ag-SR complexes were also observed in the ESI mass spectra of the final products. These Au/Ag-SR complexes are most probably produced by the decomposition of the parental [Au_*x*_Ag_44−*x*_(SR)_30_]^4−^ and [Au_44_(SR)_26_]^2−^ NCs (Fig. S16†). It should be pointed out that such Au/Ag-SR complexes were not observed in the ESI mass spectrum produced by the direct reaction of [Au_44_(SR)_26_]^2−^ and [Ag_44_(SR)_30_]^4−^ at the same Au/Ag ratio (Fig. S16†), indicating that the reaction pathways of these two processes are different. Specifically, although both parental NCs ([Au_*x*_Ag_44−*x*_(SR)_30_]^4−^ and [Au_44_(SR)_26_]^2−^, [Ag_44_(SR)_30_]^4−^ and [Au_44_(SR)_26_]^2−^) will decompose during the inter-cluster reaction, those Au atoms in [Au_*x*_Ag_44−*x*_(SR)_30_]^4−^ might not totally participate in the production of [Au_*x*_Ag_44−*x*_(SR)_27_]^3−^, which will then produce a number of Au/Ag-SR complexes (Fig. S16†). In addition, [Au_*x*_Ag_44−*x*_(SR)_27_]^3−^ can be produced by the inter-cluster reaction between [Au_*x*_Ag_44−*x*_(SR)_26_]^2−^ and [Ag_44_(SR)_30_]^4−^. Specifically, as the number of Ag atoms in [Au_*x*_Ag_44−*x*_(SR)_26_]^2−^ increases, this species can be transformed into [Au_*x*_Ag_44−*x*_(SR)_27_]^3−^, whose ESI mass spectrum is almost identical to that of the NC species produced by the direct reaction of [Ag_44_(SR)_30_]^4−^ and [Au_44_(SR)_26_]^2−^ at the same *R*_Au_44_/Ag_44__ (Fig. 4d, 2nd, 3rd, and 4th spectra). In addition, due to the similar structure between [Au_*x*_Ag_44−*x*_(SR)_27_]^3−^ and [Au_*x*_Ag_44−*x*_(SR)_26_]^2−^, no extensive Au/Ag-SR complexes were formed by the inter-cluster reaction (Fig. S17†).

**Fig. 5:**
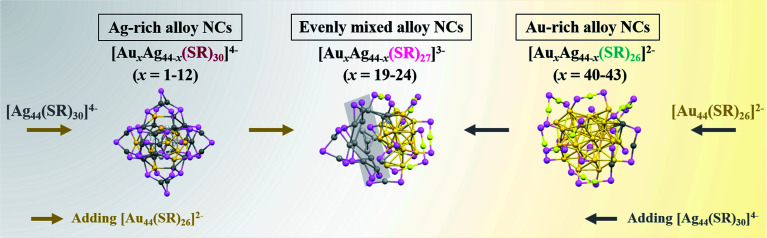
Schematic illustration of the structure evolution of Au_*x*_Ag_44–*x*_(SR)_*y*_ NCs by the inter-cluster reaction between [Ag_44_(SR)_30_]^4–^ and [Au_44_(SR)_26_]^2–^ (Color legend: yellow: Au, purple: Ag, gray: S; the remaining atoms on the ligand are omitted for clarity).

In principle, the inter-conversion among [Au_*x*_Ag_44−*x*_(SR)_30_]^4−^, [Au_*x*_Ag_44−*x*_(SR)_27_]^3−^, and [Au_*x*_Ag_44−*x*_(SR)_26_]^2−^ suggests that [Au_*x*_Ag_44−*x*_(SR)_27_]^3−^ can react with [Au_44_(SR)_26_]^2−^ to induce metal exchange while keeping the M–S framework of the parental NCs unchanged. In addition, three sets of products (*i.e.*, [Au_*x*_Ag_44−*x*_(SR)_30_]^4−^, [Au_*x*_Ag_44−*x*_(SR)_27_]^3−^, and [Au_*x*_Ag_44−*x*_(SR)_26_]^2−^) with different Au/Ag ratios can also transform among each other by adding additional metal sources of [Ag_44_(SR)_30_]^4−^ and [Au_44_(SR)_26_]^2−^ (Fig. 5). Especially, the Ag-rich [Au_*x*_Ag_44−*x*_(SR)_30_]^4−^ NCs can react with [Au_44_(SR)_26_]^2−^ to form [Au_*x*_Ag_44−*x*_(SR)_27_]^3−^, and a number of M–SR complexes will also be generated, because the Au atoms or Au-SR motifs in [Au_*x*_Ag_44−*x*_(SR)_30_]^4−^ might not participate in the alloying reaction. On the other hand, more Ag atom sources of [Ag_44_(SR)_30_]^4−^ can be provided for [Au_*x*_Ag_44−*x*_(SR)_26_]^2−^ to drive their conversion to those evenly mixed [Au_*x*_Ag_44−*x*_(SR)_27_]^3−^ NCs.

## Conclusions

In summary, we reveal the fundamentals of structural evolution from Ag_44_ to Au_44_ NCs at the molecular and atomic levels based on the reaction between [Ag_44_(SR)_30_]^4−^ and [Au_44_(SR)_26_]^2−^, which have the same metal number, but different ligand numbers and cluster structures. In particular, by increasing the feeding ratio of [Au_44_(SR)_26_]^2−^ to [Ag_44_(SR)_30_]^4−^, the structure of the alloy Au_*x*_Ag_44−*x*_ NCs evolves from Ag-rich [Au_*x*_Ag_44−*x*_(SR)_30_]^4−^ (*x* = 1–12), to evenly mixed [Au_*x*_Ag_44−*x*_(SR)_27_]^3−^ (*x* = 19–24), and finally to Au-rich [Au_*x*_Ag_44−*x*_(SR)_26_]^2−^ (*x* = 40–43) NCs, with the increase of the Au/Ag atomic ratio in the NC composition (Fig. 5). More interestingly, the three alloy products [Au_*x*_Ag_44−*x*_(SR)_30_]^4−^ (*x* = 1–12), [Au_*x*_Ag_44−*x*_(SR)_27_]^3−^ (*x* = 19–24), and [Au_*x*_Ag_44−*x*_(SR)_26_]^2−^ (*x* = 40–43) are convertible among each other through a metal exchange reaction or partial decomposition pathway during the inter-cluster reaction. This study provides molecular-level information for the inter-cluster reactions between metal NCs with the same number of metal atoms but different cluster structures. In addition, the evolution of the alloy NC products can shed fundamental light on the structure/composition-related physicochemical properties of alloy Au_*x*_Ag_44−*x*_ NCs. Moreover, the atomic-level tunability of the composition and structure of alloy NCs can provide a good means for fine tuning the electronic, optical, and catalytic properties of metal NCs, definitely adding to their acceptance in diverse fields of bioimaging, sensing, and catalysis.

## Data availability

Experimental details and additional data can be found in the ESI.†

## Author contributions

J. X. and Y. Q. proposed the ideas and supervised the project. Y. L., Y. C., and Y. Q. designed the experiments. Y. L. performed the experiments. All authors contributed to the analysis and writing.

## Conflicts of interest

There are no conflicts to declare.

## Supplementary Material

SC-013-D1SC06296D-s001
