# Supplementary material for: Revealing the composition-dependent structural evolution fundamentals of bimetallic nanoparticles through an inter-particle alloying reaction
Source: Chem Sci. 2022 Mar 25;13(16):4598–607. doi: 10.1039/d1sc06296d (PMC9020180; doi:10.1039/d1sc06296d)
Supplement: SC-013-D1SC06296D-s001 [file SC-013-D1SC06296D-s001.pdf]

Electronic Supplementary Material (ESI) for Chemical Science.
This journal is © The Royal Society of Chemistry 2022

Supporting Information

Revealing the composition-dependent structure evolution fundamentals of bimetallic nanoparticles through the inter-particle alloying reaction

Yingzheng Lin,[a,b] Yitao Cao,[b] Qiaofeng Yao,[*,b] and Jianping Xie[*,a,b]

[a] Joint School of National University of Singapore and Tianjin University, International Campus of Tianjin University, Binhai New City, Fuzhou 350207, P. R. China

[b] Department of Chemical and Biomolecular Engineering, National University of Singapore, 4 Engineering Drive 4, Singapore 117585, Singapore.

Experimental Section

Chemicals

Hydrogen tetrachloroaurate(III) trihydrate ($HAuCl_4 \cdot 3H_2O$), *para*-mercaptobenzoic acid (*p*-MBA), sodium borohydride ($NaBH_4$), cesium hydroxide hydrate ($CsOH \cdot H_2O$), sodium hydroxide (NaOH), acetic acid (HOAc) and N,N-dimethylformamide (DMF) from *Sigma Aldrich*; silver nitrate ($AgNO_3$) from *Merck*; ethanol and toluene from *Fisher* were used as-received without further purification. Ultrapure Millipore water (18.2 MΩ.cm) was used in preparation of all aqueous solutions.

Synthesis of $[Au_{44}(p\text{-MBA})_{26}]^{2-}$ nanoclusters (NCs)

$[Au_{44}(p\text{-MBA})_{26}]^{2-}$ was synthesized according to the reported method.[1] In a typical synthesis, 1.25 mL of 50 mM *p*-MBA ethanolic solution and 1.25 mL of 50 mM $HAuCl_4$ aqueous solution were added into 45 mL of ultrapure water. The pH of the solution was adjusted to 13.0 by adding 1 M NaOH solution, leading to the formation of a pale-yellow Au(I)-(*p*-MBA) complex solution. The complex solution was then stirred at 500 rpm for 30 min, before bubbling with CO for 2 min. The reaction was allowed to proceed at room temperature (25 °C) for 6 days. After 6 days, the raw product of $[Au_{44}(p\text{-MBA})_{26}]^{2-}$ was purified by adding ethanol (1/5 V/V), followed by centrifugation at 12,000 rpm for 5 mins. As the inter-cluster reaction will be carried out in dimethyl formamide (DMF) solution, $[Au_{44}(p\text{-MBA})_{26}]^{2-}$ NCs will be fully protonated. The precipitation of $[Au_{44}(p\text{-MBA})_{26}]^{2-}$ NCs was first dissolved in a DMF

solution containing 30% V/V acetic acid (HOAc), and then precipitated by adding toluene (1/4 V/V). In the second round, the precipitated $[Au_{44}(p\text{-MBA})_{26}]^{2-}$ NCs were dissolved in the DMF solution containing 10% V/V HOAc and further precipitated by adding toluene (1/4 V/V). The NC precipitate can be well dissolved in DMF for further reactions.

Synthesis of $[Ag_{44}(p\text{-MBA})_{30}]^{4-}$ NCs

The synthesis of $[Ag_{44}(p\text{-MBA})_{30}]^{4-}$ NCs was according to the reported method.[2] In a typical synthesis, 21 mL of 11.9 mM $AgNO_3$ aqueous solution was added to 12 mL of 83 mM *p*-MBA ethanolic solution. The pH of the solution was brought to 12.0 by dropping 50% V/V CsOH solution, leading to the formation of a clear Ag(I)-(*p*-MBA) complex solution. Afterwards, the complexes were reduced by adding 9 mL of $NaBH_4$ aqueous solution. The raw product of $[Ag_{44}(p\text{-MBA})_{30}]^{4-}$ NCs was obtained after 12 h of reaction and purified by adding ethanol (1/2 V/V), followed by centrifugation at 12,000 rpm for 5 mins. The protonation procedure of $[Ag_{44}(p\text{-MBA})_{30}]^{4-}$ NCs was the same as that of $[Au_{44}(p\text{-MBA})_{26}]^{2-}$ NCs.

Inter-cluster reaction

Stock solutions of $[Ag_{44}(p\text{-MBA})_{30}]^{4-}$ and $[Au_{44}(p\text{-MBA})_{26}]^{2-}$ with the same concentration of 0.01 mM NCs were firstly prepared. After that, an inter-cluster reaction with a specific Au/Ag ratio was carried out by mixing the corresponding volume ratio of $[Ag_{44}(p\text{-MBA})_{30}]^{4-}$ and $[Au_{44}(p\text{-MBA})_{26}]^{2-}$. For example, in the reaction

of $R_{Au44/Ag44}$ = 1/3, 1 mL of 0.01 mM $[Au_{44}(p\text{-MBA})_{26}]^{2-}$ solution was mixed with 3 mL of 0.01 mM $[Ag_{44}(p\text{-MBA})_{30}]^{4-}$.

Materials Characterization

UV-vis absorption spectra of the NC solution were recorded by a Shimadzu UV-1800 spectrometer. The concentration of the NC solution was determined by inductively coupled plasma - optical emission spectrometry (ICP-OES) on a Thermo Scientific iCAP 6000. Electrospray ionization mass spectrometry (ESI-MS) analysis was carried out on a Bruker microTOF-Q system in negative ion mode under the source temperature of 120 °C, dry gas flow rate of 4 L per min, nebulizer pressure of 0.4 bar, and capillary voltage of 3.5 kV. In a typical ESI-MS analysis, 0.2 mL of NCs in DMF (with a NC concentration of ~0.01 mM) was injected with a flow rate of 3 µL per min.

Supporting Figures

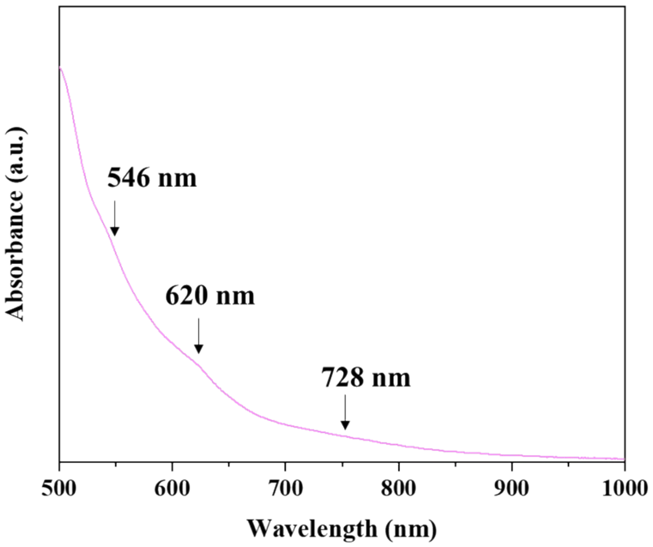

Figure S1. UV-vis absorption spectrum of the as-synthesized $[Au_{12}Ag_{32}(SR)_{30}]^{4-}$, showing three humps at 546, 620, and 728 nm.

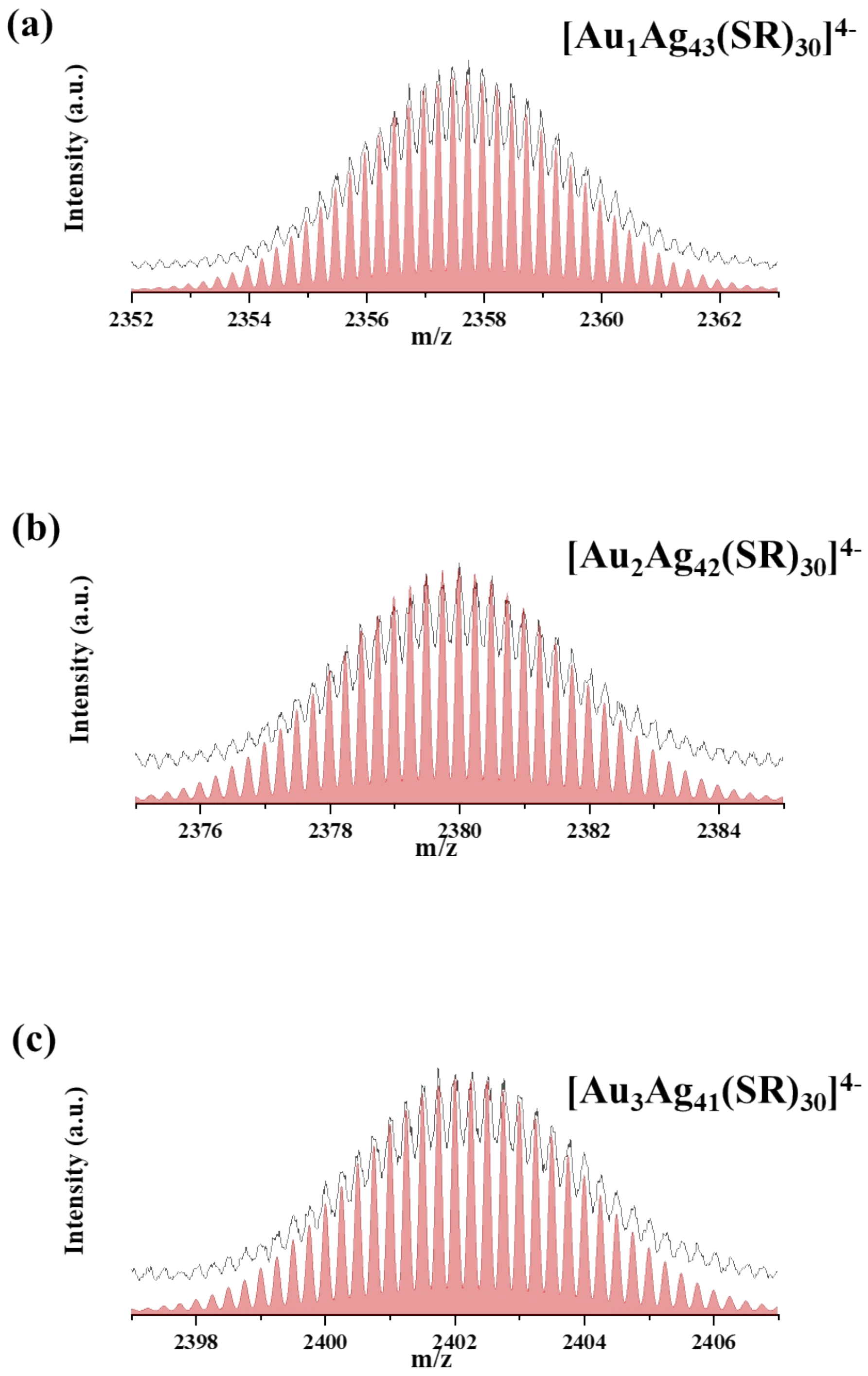
(a)
[Au1Ag43(SR)30]4-
Intensity (a.u.)
2352
2354
2356
2358
2360
2362
m/z
(b)
[Au2Ag42(SR)30]4-
Intensity (a.u.)
2376
2378
2380
2382
2384
m/z
(c)
[Au3Ag41(SR)30]4-
Intensity (a.u.)
2398
2400
2402
2404
2406
m/z

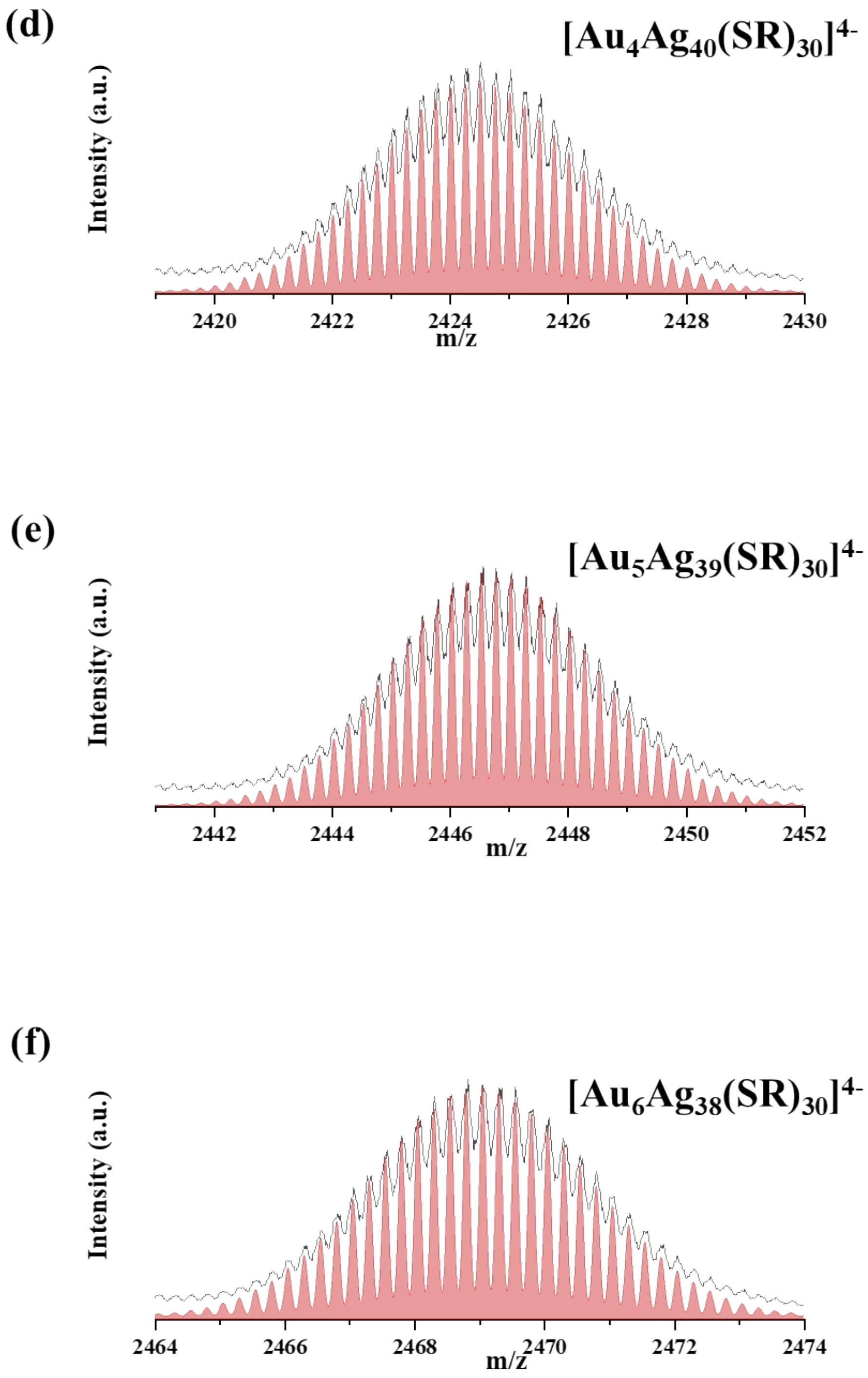
(d)
$[Au_4Ag_{40}(SR)_{30}]^{4-}$
Intensity (a.u.)
2420
2422
2424
2426
2428
2430
m/z
(e)
$[Au_5Ag_{39}(SR)_{30}]^{4-}$
Intensity (a.u.)
2442
2444
2446
2448
2450
2452
m/z
(f)
$[Au_6Ag_{38}(SR)_{30}]^{4-}$
Intensity (a.u.)
2464
2466
2468
2470
2472
2474
m/z

(g)

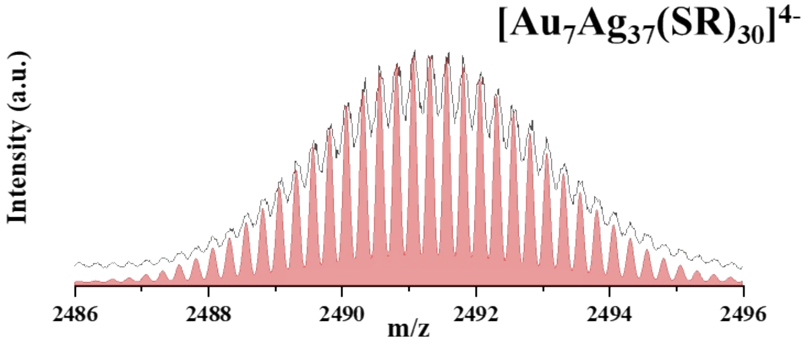
$[Au_7Ag_{37}(SR)_{30}]^{4-}$
Intensity (a.u.)
2486
2488
2490
2492
2494
2496
m/z

(h)

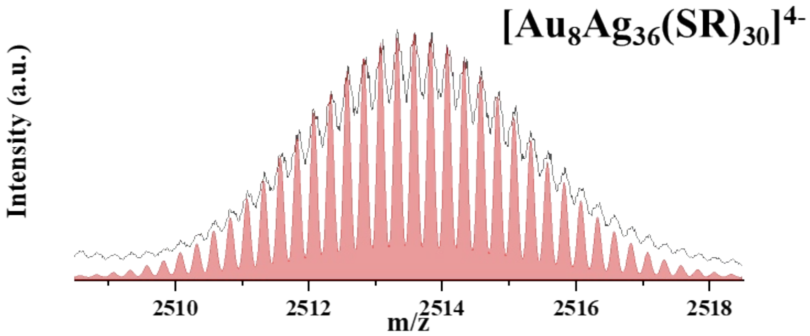
$[Au_8Ag_{36}(SR)_{30}]^{4-}$
Intensity (a.u.)
2510
2512
2514
2516
2518
m/z

(i)

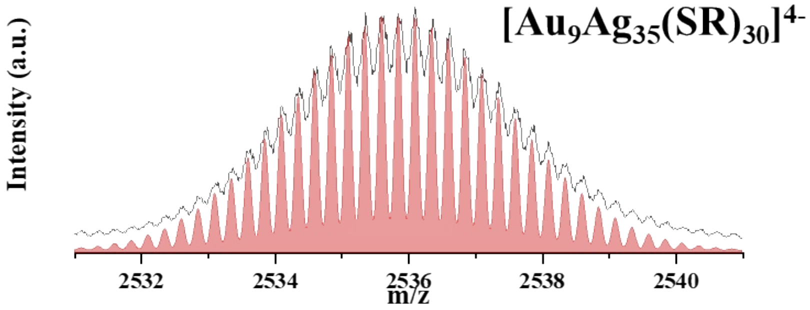
$[Au_9Ag_{35}(SR)_{30}]^{4-}$
Intensity (a.u.)
2532
2534
2536
2538
2540
m/z

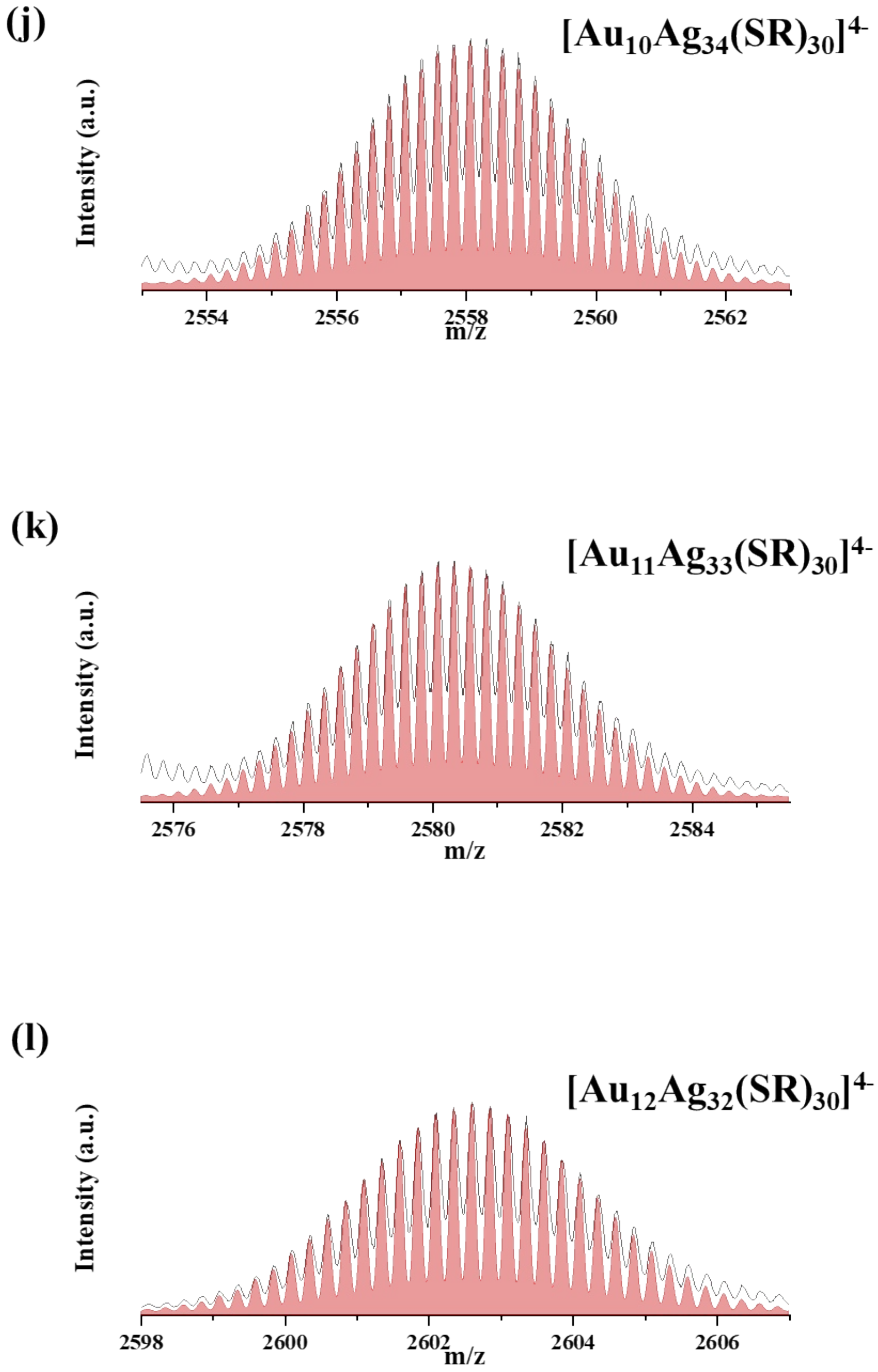

Figure S2. Experimental (black lines) and simulated (red lines) isotope patterns of $[Au_xAg_{44\text{-}x}(p\text{-MBA})_{30}]^{4-}$ (x = 1-12) NCs.

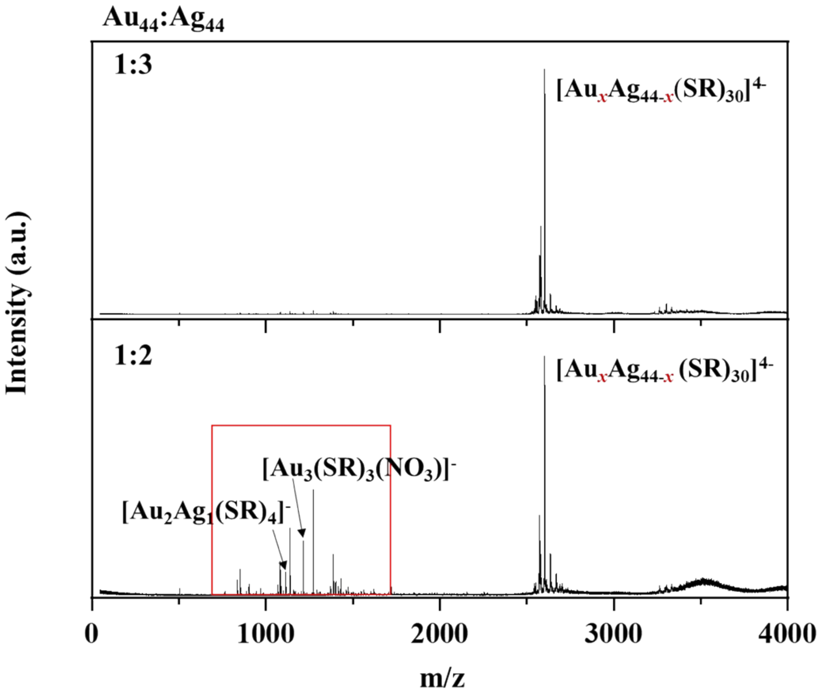

Figure S3. ESI mass spectra of alloy Au/Ag NCs synthesized at $R_{\mathrm{Au44/Ag44}}$ = 1/3 and 1/2.

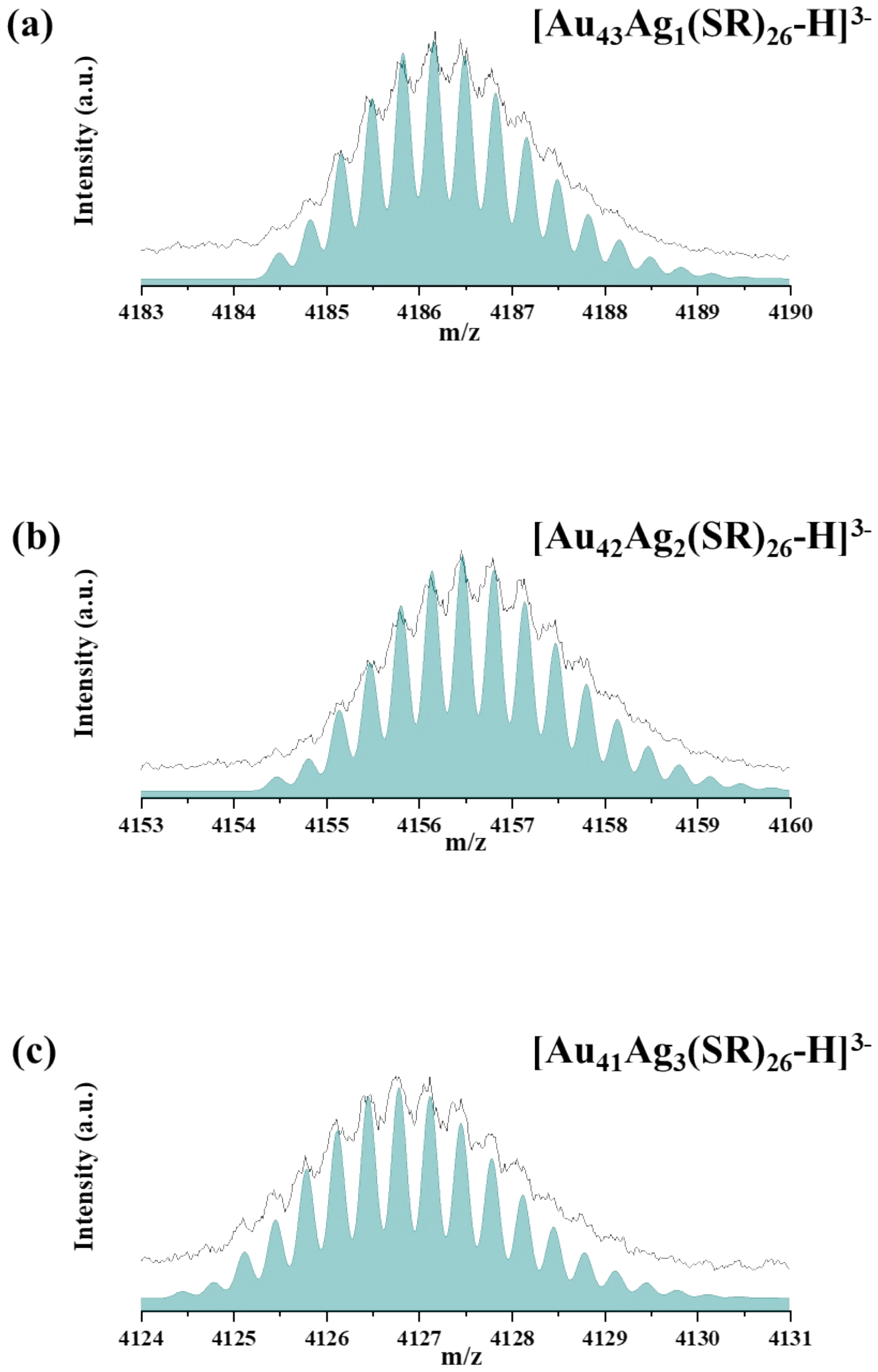
(a)
$[Au_{43}Ag_1(SR)_{26}\text{-}H]^{3-}$
Intensity (a.u.)
4183 4184 4185 4186 4187 4188 4189 4190
m/z
(b)
$[Au_{42}Ag_2(SR)_{26}\text{-}H]^{3-}$
Intensity (a.u.)
4153 4154 4155 4156 4157 4158 4159 4160
m/z
(c)
$[Au_{41}Ag_3(SR)_{26}\text{-}H]^{3-}$
Intensity (a.u.)
4124 4125 4126 4127 4128 4129 4130 4131
m/z

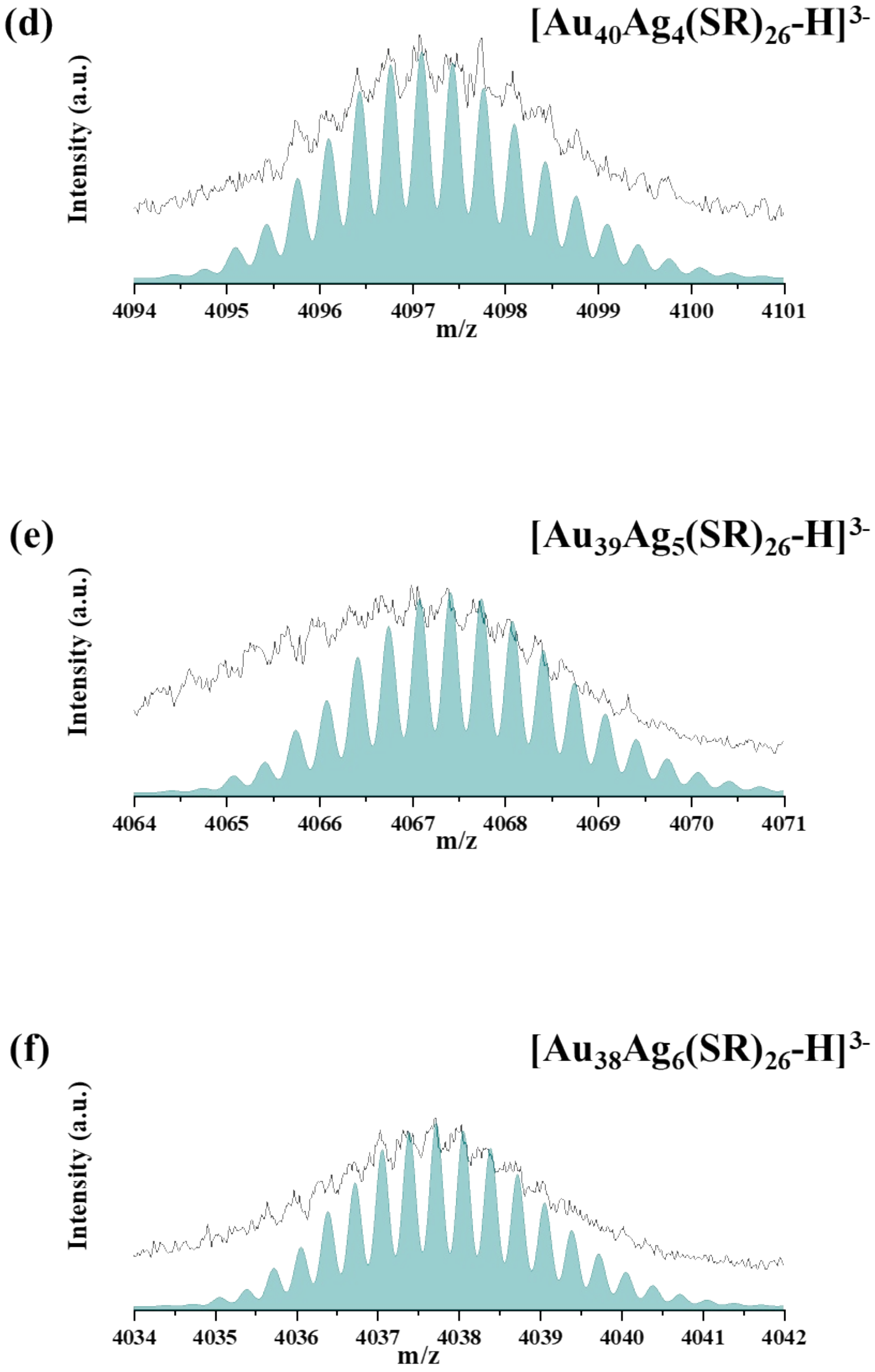
(d)
[Au40Ag4(SR)26-H]3-
Intensity (a.u.)
4094 4095 4096 4097 4098 4099 4100 4101
m/z
(e)
[Au39Ag5(SR)26-H]3-
Intensity (a.u.)
4064 4065 4066 4067 4068 4069 4070 4071
m/z
(f)
[Au38Ag6(SR)26-H]3-
Intensity (a.u.)
4034 4035 4036 4037 4038 4039 4040 4041 4042
m/z

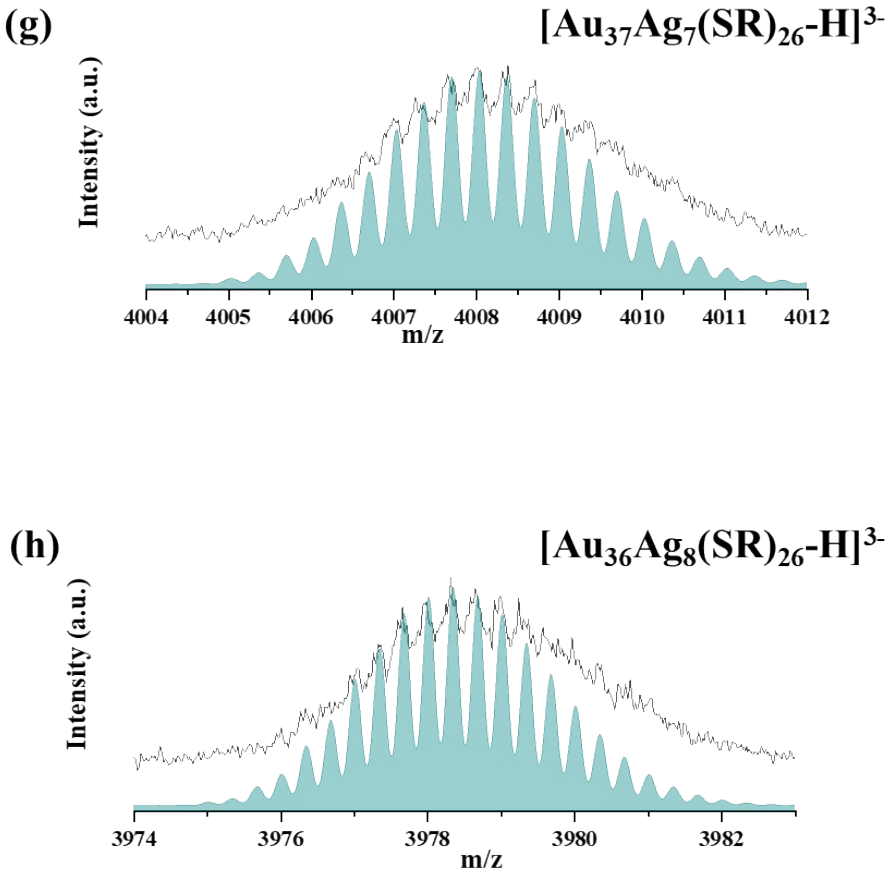

Figure S4. Experimental (black lines) and simulated (green lines) isotope patterns of $[Au_xAg_{44-x}(SR)_{26} - H]^{3-}$ (x = 36-43) NCs.

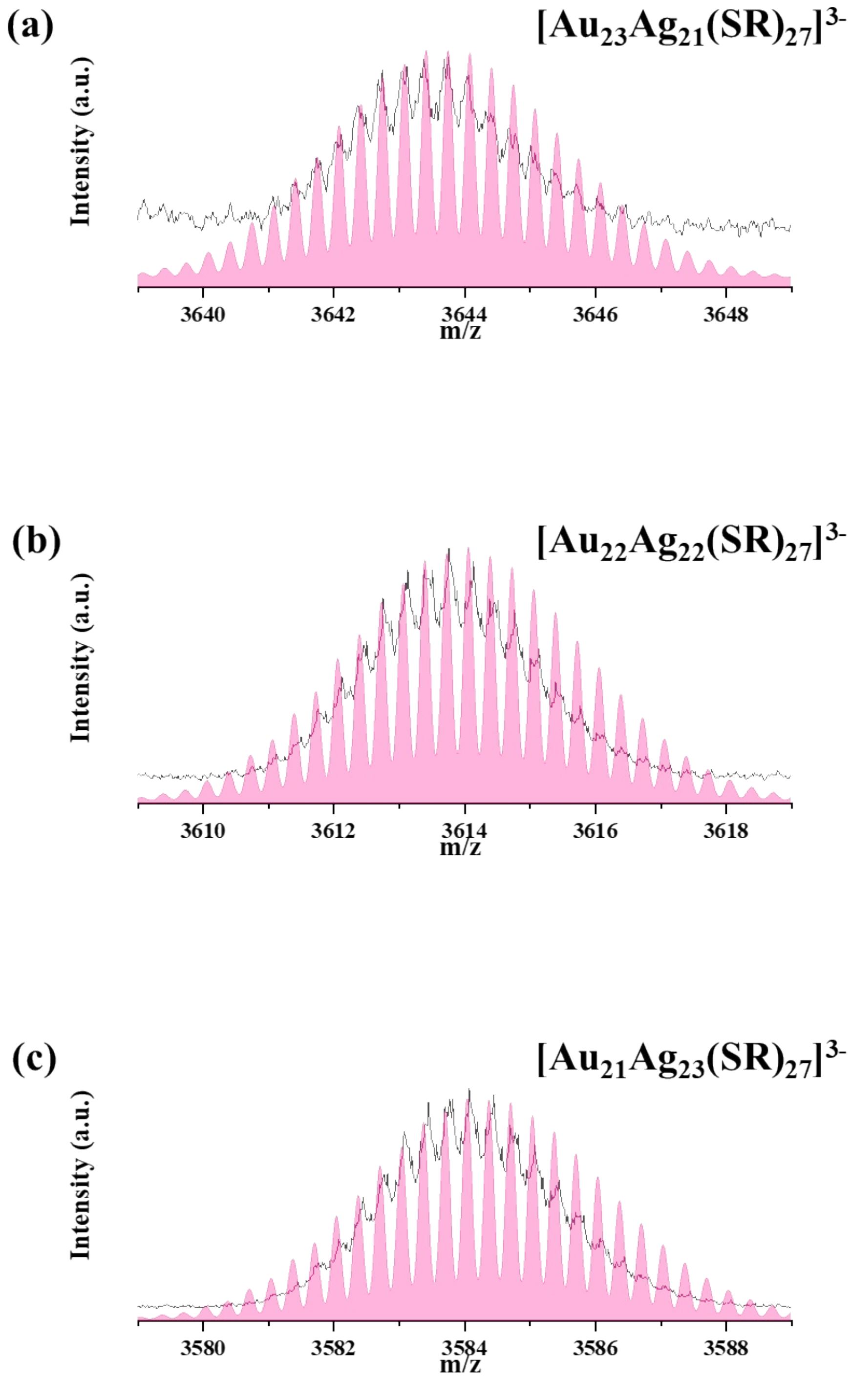
(a)
$[Au_{23}Ag_{21}(SR)_{27}]^{3-}$
Intensity (a.u.)
3640
3642
3644
3646
3648
m/z
(b)
$[Au_{22}Ag_{22}(SR)_{27}]^{3-}$
Intensity (a.u.)
3610
3612
3614
3616
3618
m/z
(c)
$[Au_{21}Ag_{23}(SR)_{27}]^{3-}$
Intensity (a.u.)
3580
3582
3584
3586
3588
m/z

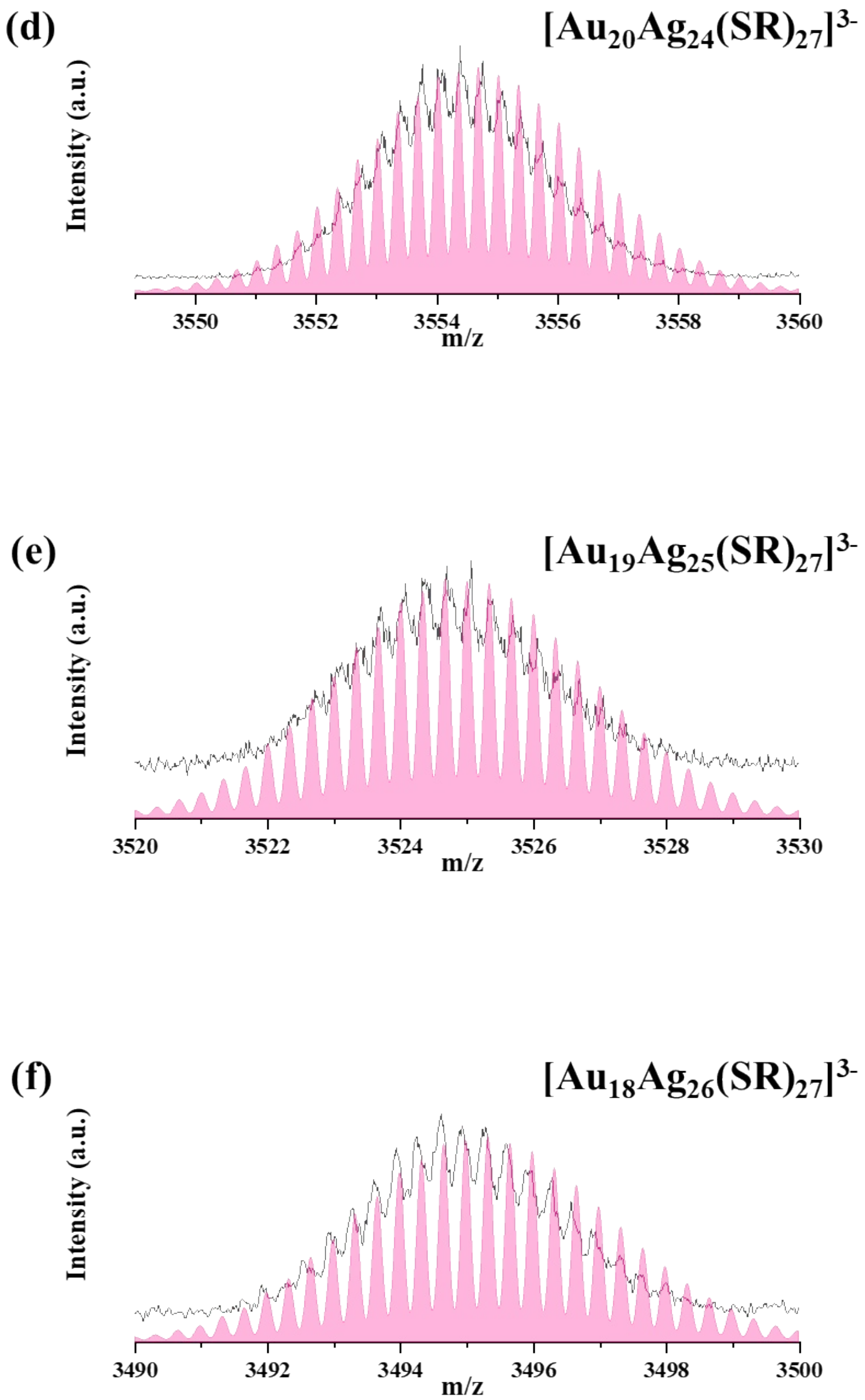

Figure S5. Experimental (black lines) and simulated (pink lines) isotope patterns of $[Au_xAg_{44-x}(SR)_{27}]^{3-}$ (x = 18-23) NCs.

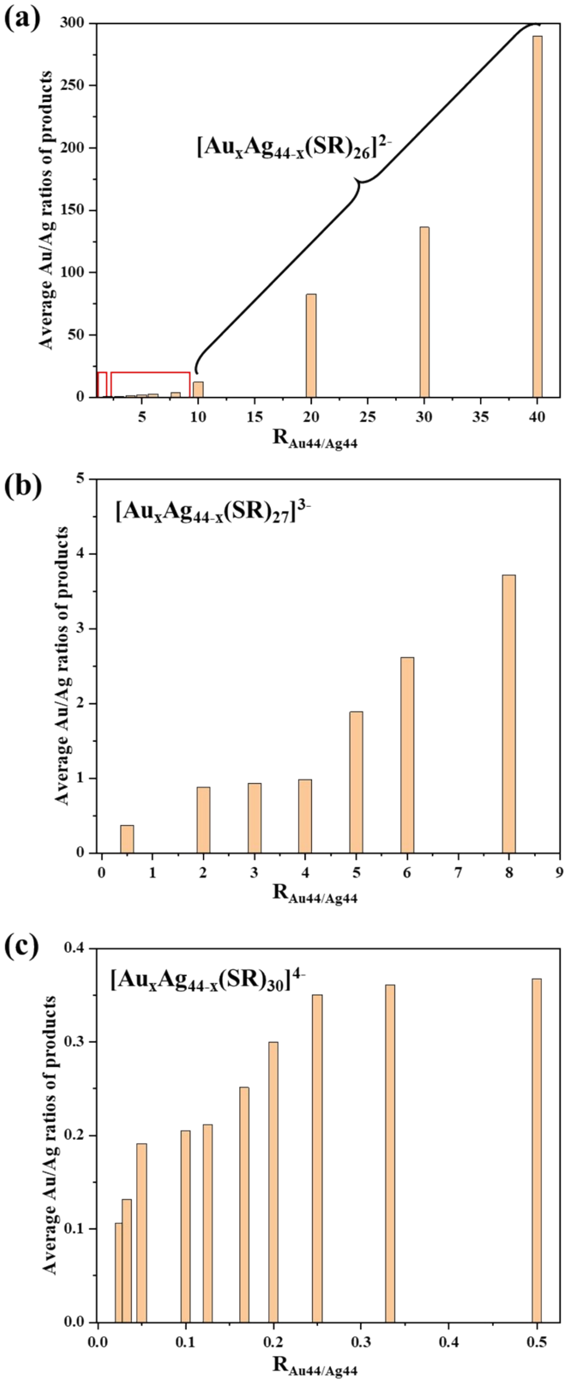

Figure S6. (a) The correlation between feeding Au_{44}/Ag_{44} ratios and average Au/Ag ratios of products. (b) The zoom-in view of the boxed area (with a feeding Au_{44}/Ag_{44} ratios of 0 to 0.5). (c) The zoom-in view of the boxed area (with a feeding Au_{44}/Ag_{44} ratios of 0.5 to 8).

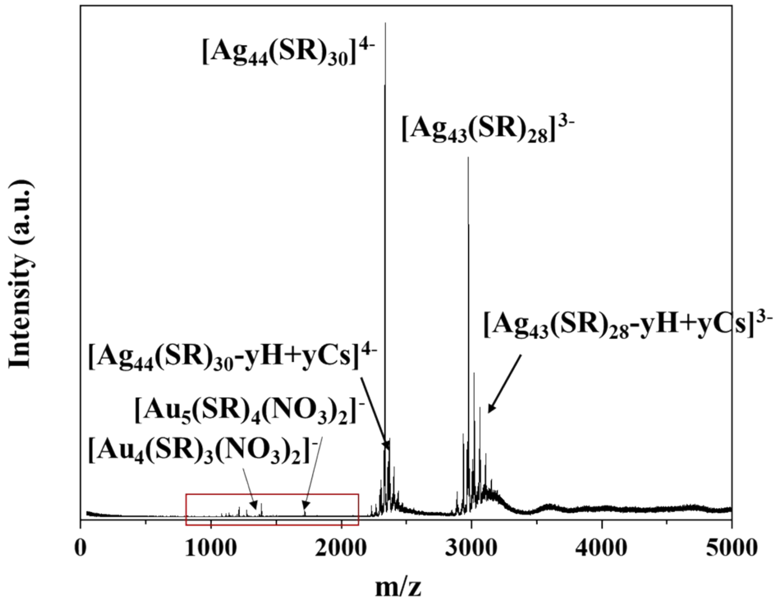

Figure S7. ESI mass spectrum of the reaction between $[Au_{44}(SR)_{26}]^{2-}$ and $[Ag_{44}(SR)_{30}]^{4-}$ ($R_{Au44/Ag44}$ = 1/3) at the reaction time (*t*) of 2 min.

(a)

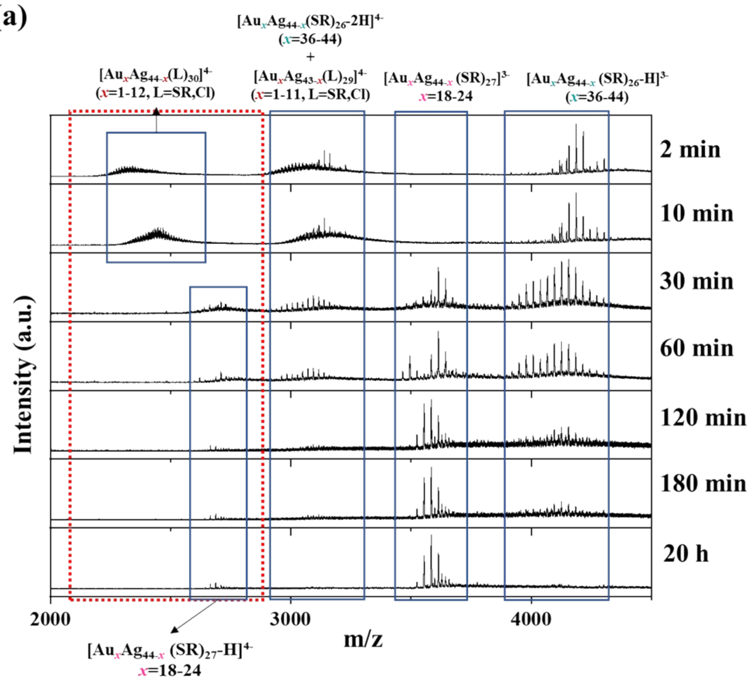

[AuxAg44-x(SR)26-2H]4-
(x=36-44)
+
[AuxAg44-x(L)30]4-
(x=1-12, L=SR,Cl)
[AuxAg43-x(L)29]4-
(x=1-11, L=SR,Cl)
[AuxAg44-x (SR)27]3-
x=18-24
[AuxAg44-x (SR)26-H]3-
(x=36-44)
2 min
10 min
30 min
60 min
120 min
180 min
20 h
Intensity (a.u.)
2000
3000
4000
m/z
[AuxAg44-x (SR)27-H]4-
x=18-24

(b)

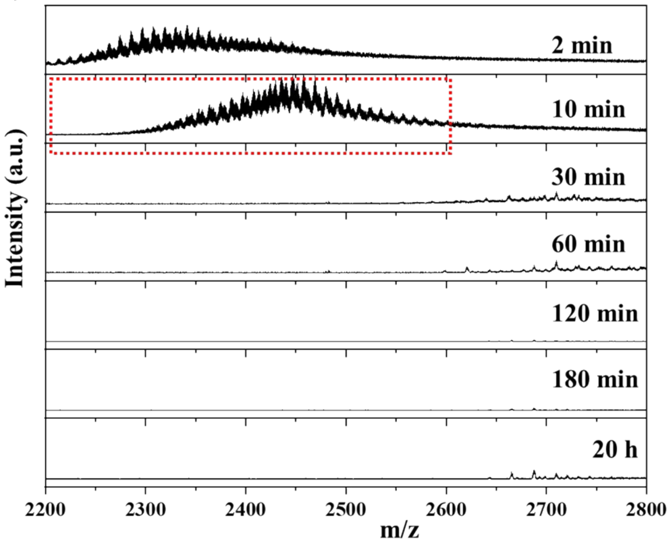

2 min
10 min
30 min
60 min
120 min
180 min
20 h
Intensity (a.u.)
2200
2300
2400
2500
2600
2700
2800
m/z

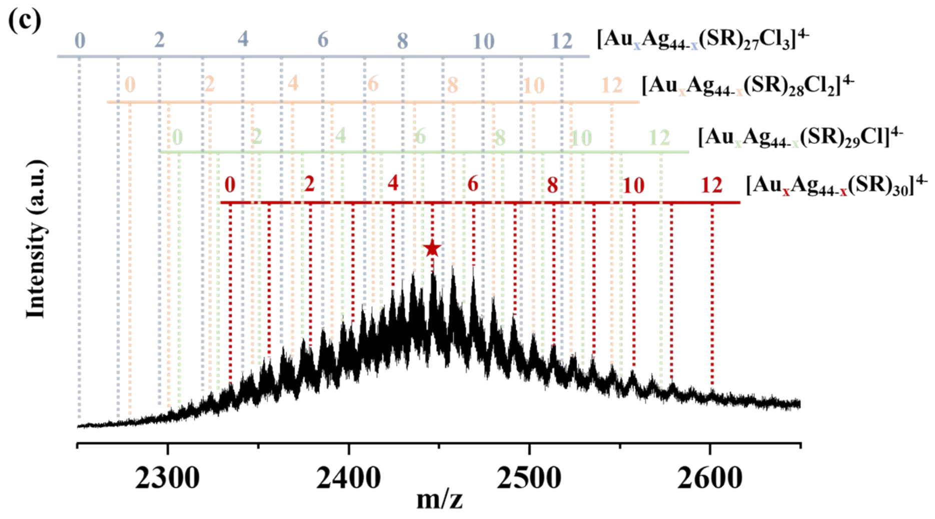

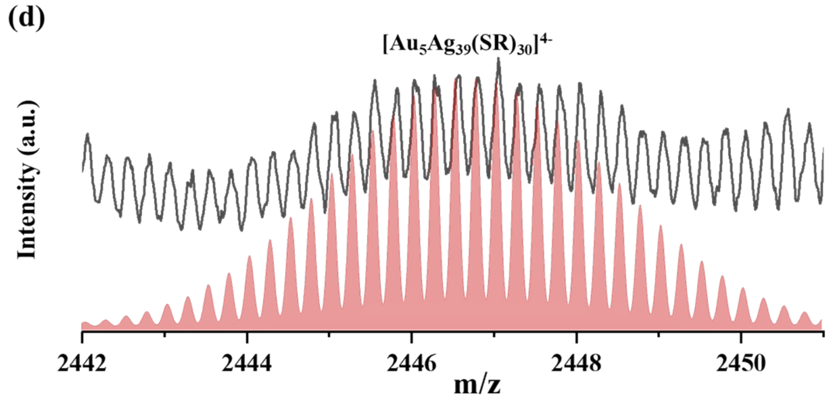

Figure S8. Time-course ESI mass spectra of the reaction between $[Au_{44}(SR)_{26}]^{2-}$ and $[Ag_{44}(SR)_{30}]^{4-}$ ($R_{Au44/Ag44}$ = 3/1) in the *m/z* range of (a) 2000-4500 and (b) 2200-2800. (c) The formula assignment for those peaks in the *m/z* range of 2200-2800 at t = 10 min. (d) Experimental (black lines) and simulated (red lines) isotope patterns of $[Au_5Ag_{39}(SR)_{30}]^{4-}$ (this peak was selected from (c), labeled by a red asterisk).

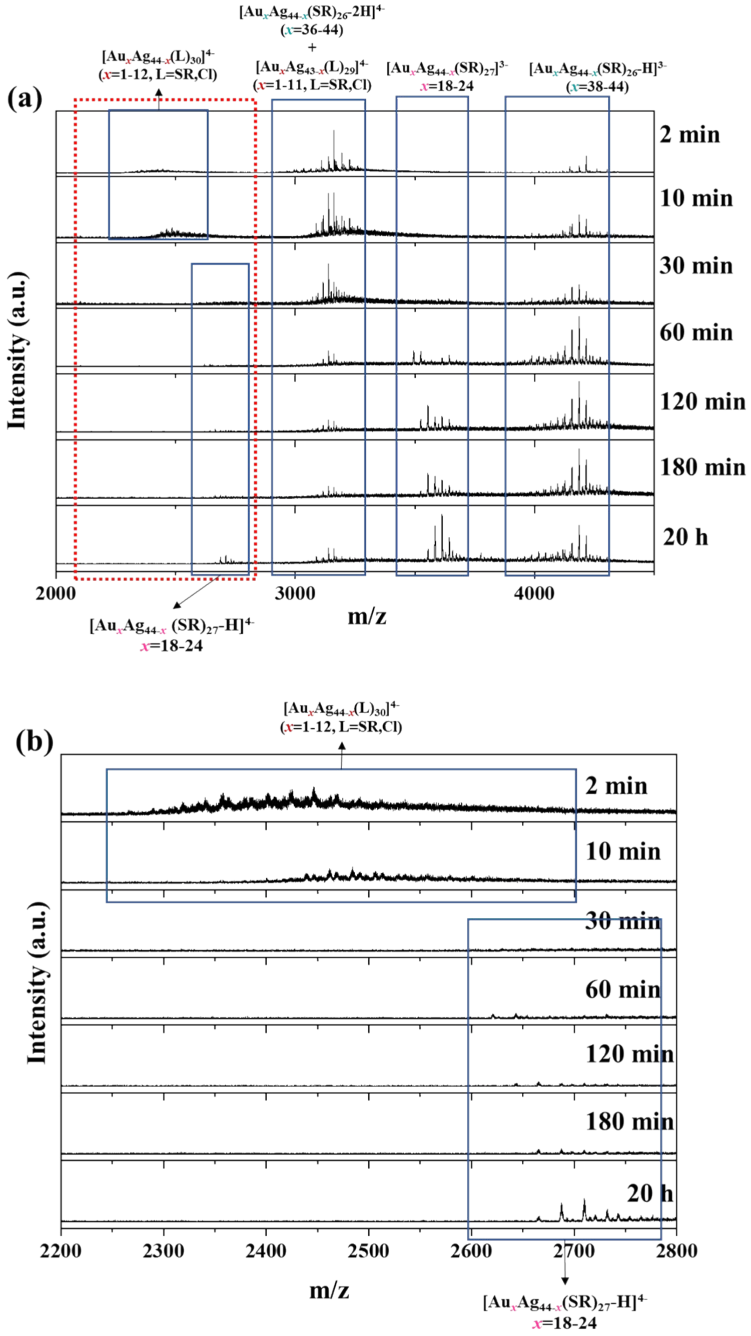

Figure S9. Time-course ESI mass spectra of the reaction between $[Au_{44}(SR)_{26}]^{2-}$ and $[Ag_{44}(SR)_{30}]^{4-}$ ($R_{Au44/Ag44}$ = 6/1) at the m/z range of (a) 2000-4500 and (b) 2200-2800.

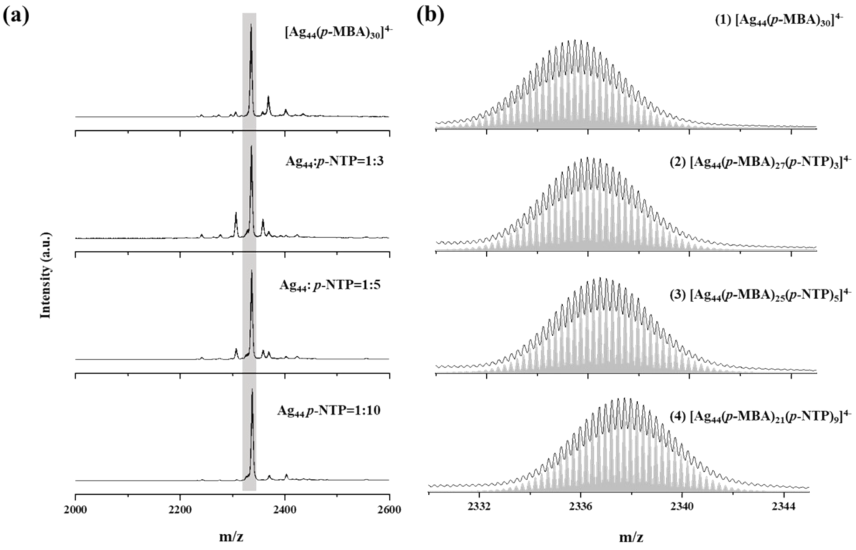

Figure S10. Ligand exchange of $[Ag_{44}(p\text{-MBA})_{30}]^{4-}$ with *p*-NTP at different ratios. (a) The mass spectrum in *m/z* range from 2000 to 2600. (b) The zoom-in of the box area of each corresponding panel with *m/z* from 2330 to 2344.

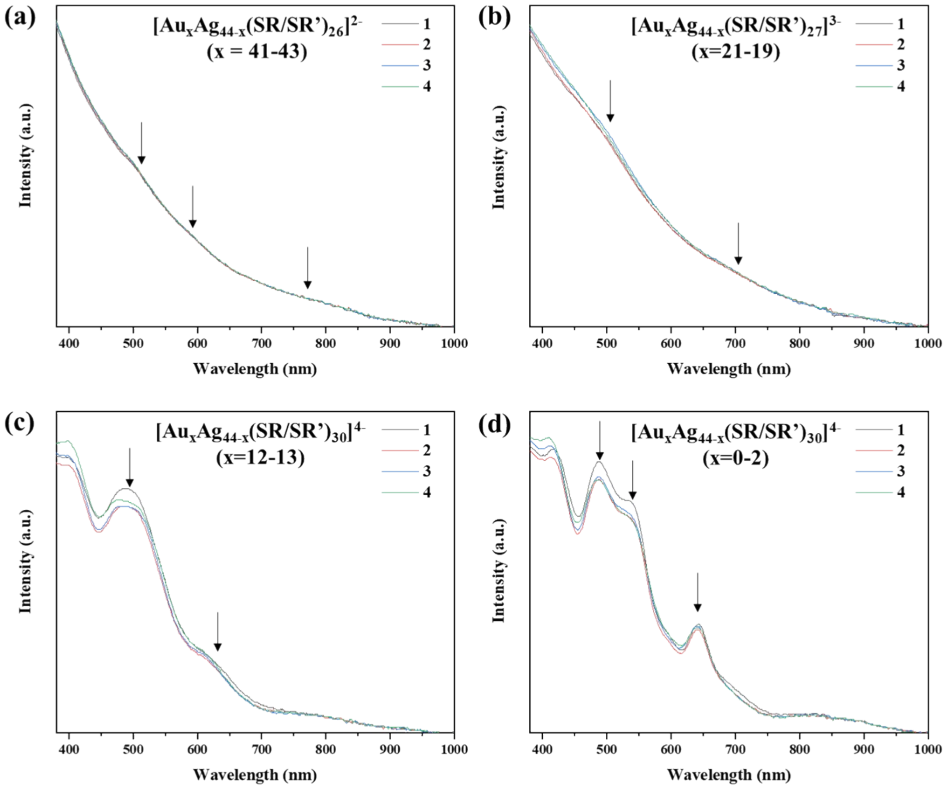

Figure S11. UV-Vis spectra of intercluster reactions between bi-ligand $[Ag_{44}(p\text{-}MBA/p\text{-}NTP)_{30}]^{4-}$ and $[Au_{44}(p\text{-}MBA)_{26}]^{2-}$ at different ratios to generate bi-ligand alloy NCs of (a) $[Au_xAg_{44-x}(SR/SR')_{26}]^{2-}$ (x = 41-43), (b) $[Au_xAg_{44-x}(SR/SR')_{27}]^{3-}$ (x = 19-21), (c) $[Au_xAg_{44-x}(SR/SR')_{30}]^{4-}$ (x = 12-13), and (d) $[Au_xAg_{44-x}(SR/SR')_{30}]^{4-}$ (x = 0-2).

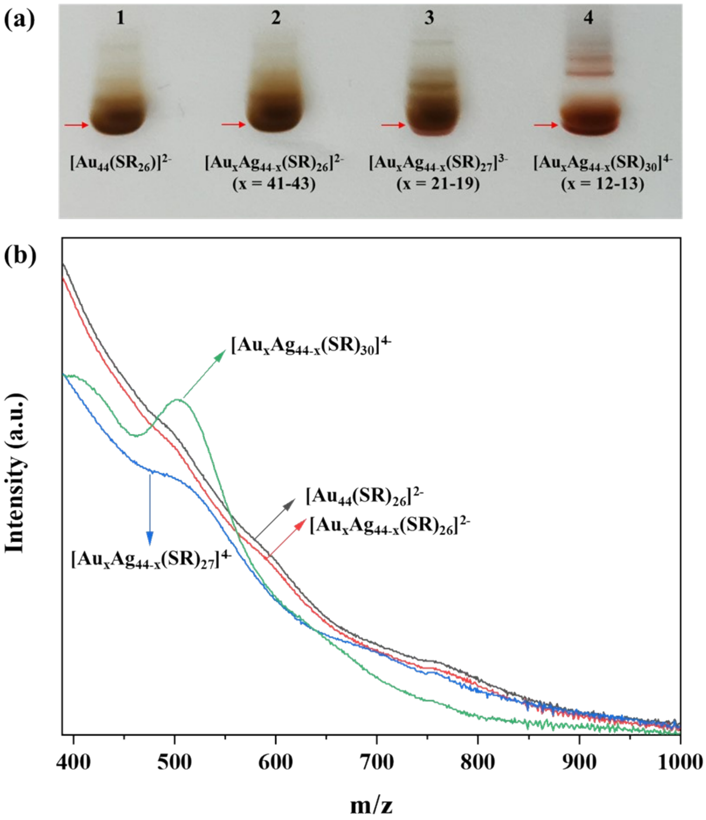

Figure S12. (a) Digital photo of the PAGE bands of the Au NCs and the Au/Ag alloy NCs. (b) The UV-Vis spectrum of $[Au_{44}(SR)_{26}]^{2-}$, $[Au_xAg_{44-x}(SR)_{26}]^{2-}$, $[Au_xAg_{44-x}(SR)_{27}]^{3-}$and $[Au_xAg_{44-x}(SR)_{30}]^{4-}$ after PAGE separation.

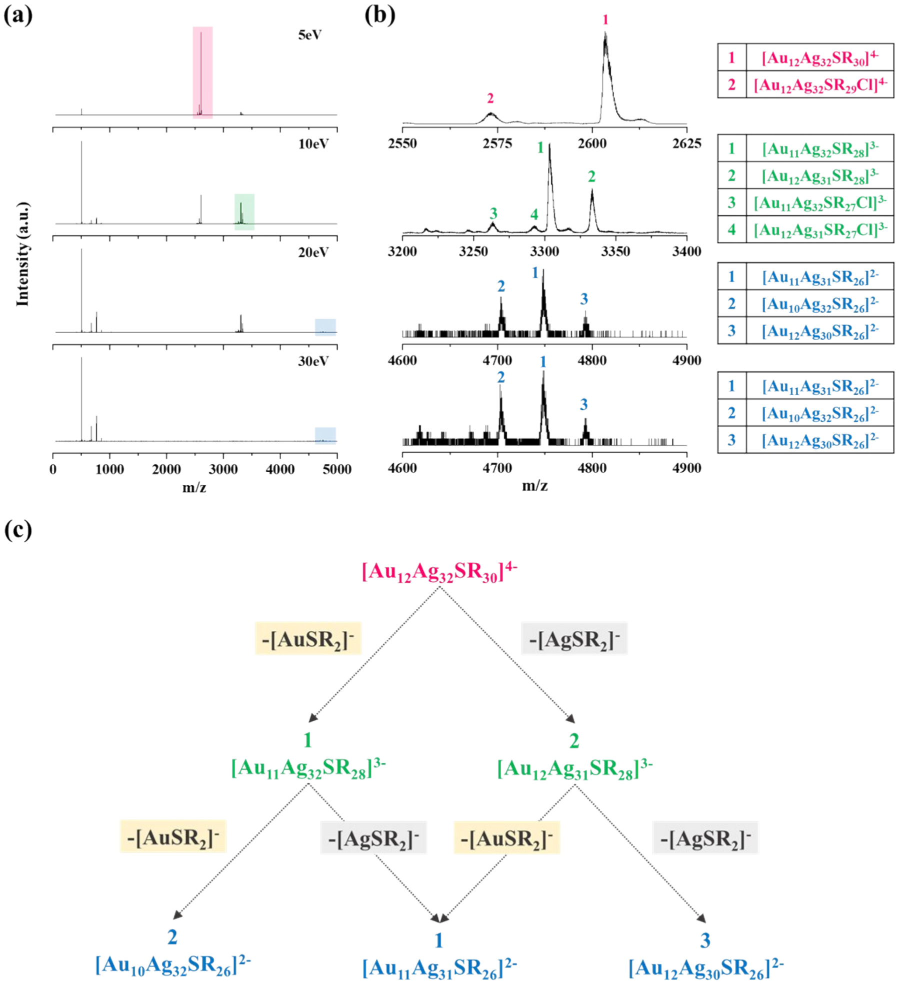

Figure S13. (a) Tandem mass spectra of $[Au_{12}Ag_{32}(SR)_{30}]^{4-}$ with m/z = 2602.5 at varied collision energies. (b) Zoom-in spectra of the boxed area in corresponding panel. (c) fragmentation process of $[Au_{12}Ag_{32}(SR)_{30}]^{4-}$ with m/z = 2602.5 in tandem mass spectrometry analysis.

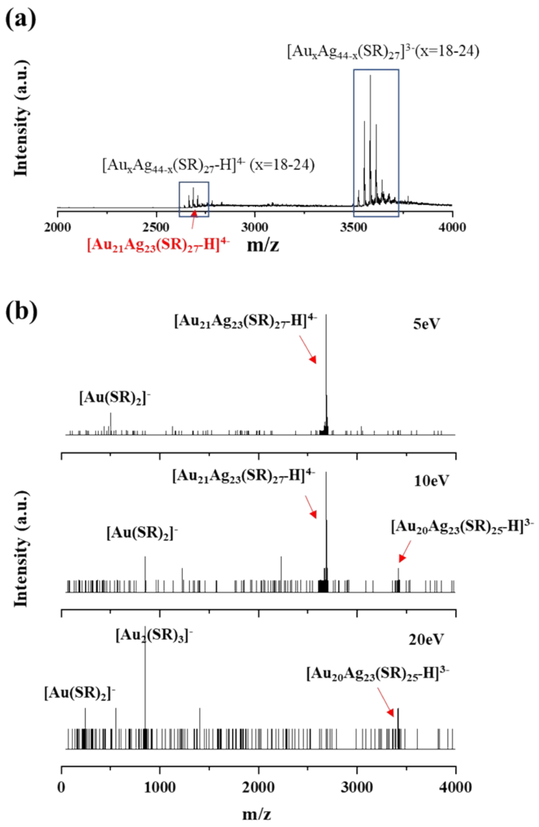

Figure S14. (a) ESI mass spectrum of $[Au_xAg_{44-x}(SR)_{27}]^{3-}$ NCs. (b) Tandem mass spectra of $[Au_{21}Ag_{23}(SR)_{27}\text{-H}]^{4-}$ with m/z = 2687.75 at varied collision energies.

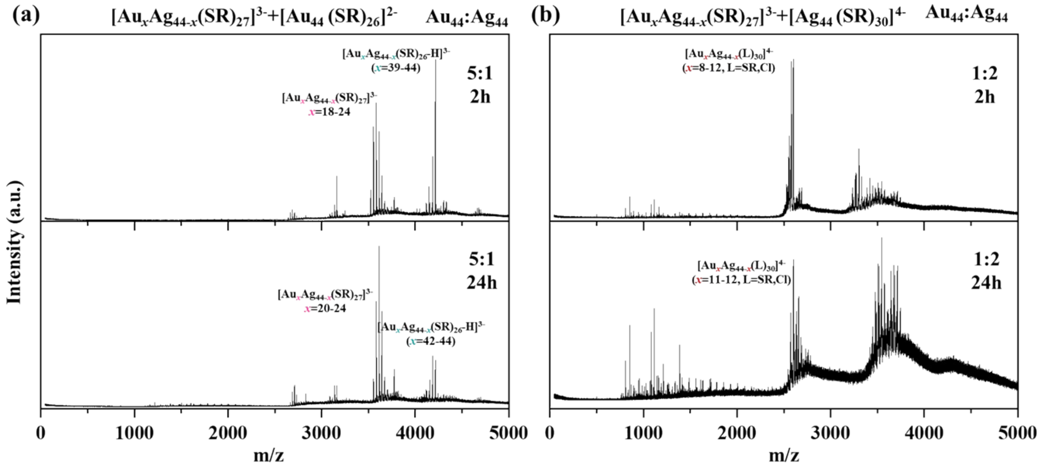

Figure S15. (a) Broad-range ESI mass spectra of the reactions between $[Au_xAu_{44-x}(SR)_{27}]^{3-}$ and $[Au_{44}(SR)_{26}]^{2-}$ ($t = 2$ and 24 h, $R_{Au44/Ag44} = 5/1$). (b) Broad-range ESI mass spectra of the reactions between $[Au_xAu_{44-x}(SR)_{27}]^{3-}$ and $[Ag_{44}(SR)_{30}]^{2-}$ ($t = 2$ and 24 h, $R_{Au44/Ag44} = 1/2$).

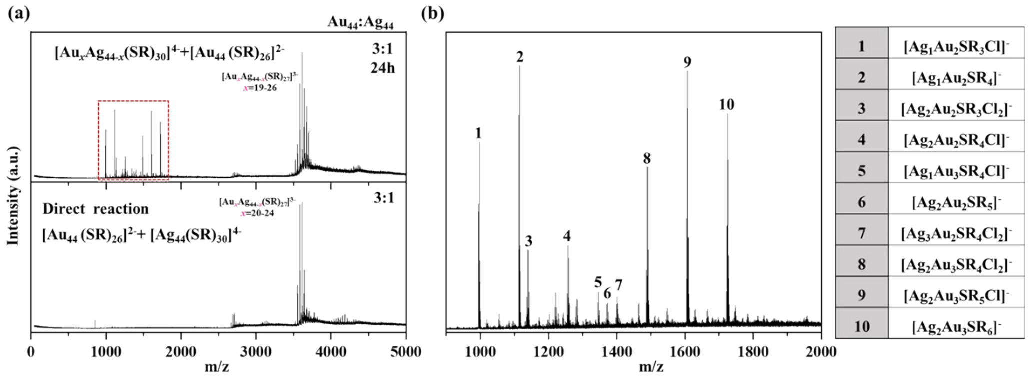

Figure S16. (a) Broad-range ESI mass spectra of the final products of inter-cluster reaction between $[Au_xAg_{44-x}(SR)_{30}]^{4-}$ and $[Au_{44}(SR)_{26}]^{2-}$ (top panel), and direct reaction between $[Ag_{44}(SR)_{30}]^{4-}$ and $[Au_{44}(SR)_{26}]^{2-}$ (bottom panel) at $R_{Au44/Ag44} = 3/1$. (b) Zoom-in ESI mass spectra of the inter-cluster reaction between $[Au_xAg_{44-x}(SR)_{30}]^{4-}$ and $[Au_{44}(SR)_{26}]^{2-}$ at the *m/z* range of 900-2000. The right panel in (b) shows the molecular formulae of the labeled peaks.

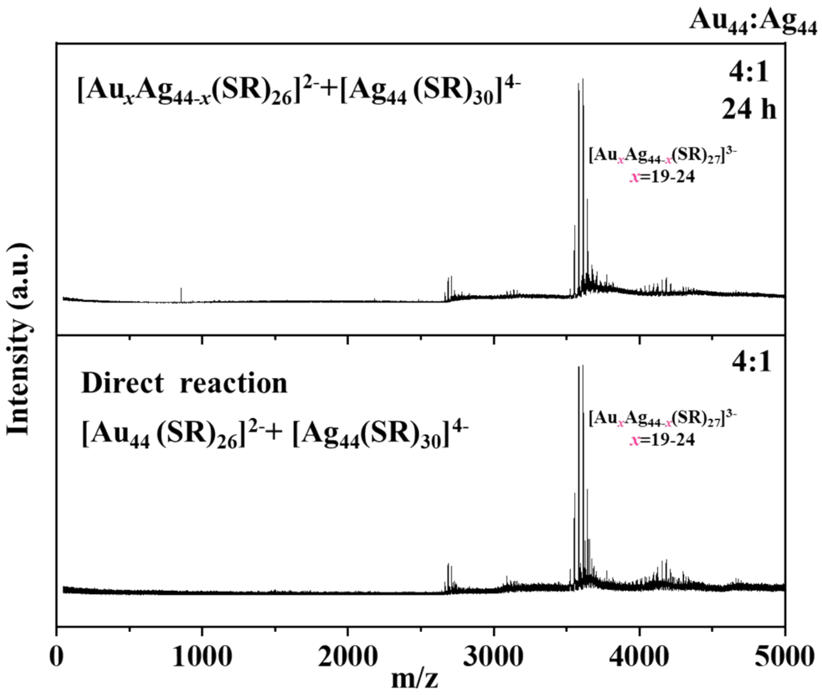

Figure S17. (a) ESI mass spectra of the final products of the inter-cluster reaction between $[Au_xAg_{44\text{-}x}(SR)_{26}]^{2-}$ and $[Ag_{44}(SR)_{30}]^{4-}$ (top panel), and direct reaction between $[Ag_{44}(SR)_{30}]^{4-}$ and $[Au_{44}(SR)_{26}]^{2-}$ at $R_{\mathrm{Au44/Ag44}} = 4/1$.

Table S1 Average feeding valence electrons in the inter-cluster alloy reactions and the corresponding alloy NCs products

Au_{44}:Ag_{44}	Average feeding electrons	Alloy NCs	Valence electrons
1:40	18.0		
1:30	18.1		
1:20	18.1		
1:10	18.2		
1:8	18.2	$[Au_xAg_{44-x}(SR)_{30}]^{4-}$ (x=1-12)	18
1:6	18.3		
1:5	18.3		
1:4	18.4		
1:3	18.5		
1:2	18.7		
2:1	19.3		
3:1	19.5		
4:1	19.6	$[Au_xAg_{44-x}(SR)_{27}]^{3-}$ (x=18-24)	
5:1	19.7		
6:1	19.7		20
8:1	19.8		
10:1	19.8		
20:1	19.9	$[Au_xAg_{44-x}(SR)_{26}]^{2-}$ (x=40-44)	
30:1	19.9		
40:1	20.0		

Reference

1 Q. Yao, X. Yuan, V. Fung, Y. Yu, D. T. Leong, D. E. Jiang, J. Xie, *Nat. Commun.* **2017**, *8*, 927.

2 A. Desireddy, B. E. Conn, J. Guo, B. Yoon, R. N. Barnett, B. M. Monahan, K. Kirschbaum, W. P. Griffith, R. L. Whetten, U. Landman, T. P. Bigioni, *Nature* **2013**, *501*, 399
